# Sex Differences in Cardiac Troponin Trajectories Over the Life Course

**DOI:** 10.1161/CIRCULATIONAHA.123.064386

**Published:** 2023-04-28

**Authors:** Marie de Bakker, Atul Anand, Martin Shipley, Takeshi Fujisawa, Anoop S.V. Shah, Isabella Kardys, Eric Boersma, Eric J. Brunner, Nicholas L. Mills, Dorien M. Kimenai

**Affiliations:** 1 British Heart Foundation Centre for Cardiovascular Science, University of Edinburgh, United Kingdom (T.F., A.A., N.L.M., D.M.K.).; 2 Usher Institute, University of Edinburgh, United Kingdom (N.L.M.).; 3 Department of Cardiology, Erasmus MC Cardiovascular Institute, University Medical Center Rotterdam, the Netherlands (M.d.B., I.K., E.B.).; 4 Department of Epidemiology and Public Health, University College London, United Kingdom (M.S., E.J.B.).; 5 Department of Non-Communicable Disease, London School of Hygiene and Tropical Medicine, United Kingdom (A.S.V.S.).

**Keywords:** cardiovascular diseases, population health, risk assessment, sex, troponin

## Abstract

Cardiac troponin concentrations are lower in women than men. We examined whether age- and risk factor-related changes in cardiac troponin over the life course differ by sex and if the trajectory of cardiac troponin was informative in respect of cardiovascular outcomes in women and men in the general population.

**Methods:**

In the Whitehall II cohort, high-sensitivity cardiac troponin I concentrations were measured on 3 occasions over a 15-year period. Using linear mixed-effects models, the sex-specific trajectories of cardiac troponin were evaluated, and the relationship with conventional cardiovascular risk factors determined. Using multistate joint models, the association between sex-specific trajectories of cardiac troponin and a composite outcome of nonfatal myocardial infarction, nonfatal stroke, or cardiovascular death was evaluated.

**Results:**

In 2142 women and 5151 men (mean, 58±7 and 57±7 years of age, respectively), there were 177 (8.3%) and 520 (10.1%) outcome events, respectively, during a median follow-up of 20.9 (25th to 75th percentile, 15.8–21.3) years. Cardiac troponin concentrations were persistently lower in women than in men (median baseline concentration: 2.4 [25th to 75th percentile, 1.7–3.6] ng/L versus 3.7 [25th to 75th percentile, 2.6–5.8] ng/L, respectively, *P*<0.001), with women exhibiting a relatively larger increase with advancing age as compared with men (*P*_*interaction*_<0.001). Apart from age, a significant and divergent interaction with sex was found for the association between cardiac troponin and body mass index (BMI) (*P*_*interaction*_=0.008) and diabetes (*P*_*interaction*_=0.003). During follow-up, cardiac troponin concentrations were associated to the outcome in both women and men (adjusted hazard ratio per 2-fold difference [95% CI, 1.34 (1.17–1.52) and 1.30 (1.21–1.40), respectively], *P*_*interaction*_*=*0.752). The slope of cardiac troponin was significantly associated with the outcome in women, but not in men (adjusted hazard ratio [95% CI, 2.70 (1.01–7.33) and 1.31 (0.62–2.75), respectively], *P*_*interaction*_*=*0.250).

**Conclusions:**

Trajectories of cardiac troponin differ between women and men in the general population, with differing associations to conventional risk factors and cardiovascular outcomes. Our findings highlight the importance of a sex-specific approach when serial cardiac troponin testing is applied for cardiovascular risk prediction.

**Clinical Perspective**

**What is New?**

- We report the sex-specific trajectories of high-sensitivity cardiac troponin I over the life course, and evaluated if the trajectory of cardiac troponin was informative in respect of cardiovascular outcomes in women and men in the general population.
- Cardiac troponin concentrations in women lag around a decade behind men, but women exhibit a steeper trajectory with advancing age.
- Trajectories of cardiac troponin differ between women and men in the general population, with differing associations to conventional risk factors and cardiovascular outcomes.

**What Are the Clinical Implications?**

- Use of the same cardiac troponin thresholds to guide risk of future cardiovascular events in women and men would not provide equivalent prediction.
- Our findings highlight the importance of a sex-specific approach when serial cardiac troponin testing is applied for cardiovascular risk prediction.

Sex differences in preventative cardiovascular medicine are under explored. Recent evidence suggests that the distribution and burden of coronary atherosclerosis is similar in women and men, but that disease onset is delayed by a decade in women.^1^ It is important that cardiovascular risk estimation systems recognize this important difference between women and men to ensure approaches to the prevention of cardiovascular disease are equitable.

Cardiac troponin is a promising biomarker that may improve cardiovascular risk prediction in the general population.^2–10^ With the introduction of high-sensitivity assays, differences in cardiac troponin levels in apparently healthy women and men became evident.^11–13^ Although it is now established that cardiac troponin concentrations are lower in women than men in the general population,^11–14^ whether changes in cardiac troponin concentration over the life course differ in women and men and how this impacts on cardiovascular risk prediction are unknown. We hypothesize that increases in cardiac troponin with age will be delayed in women compared to men. Insights into sex-specific trajectories and how these are influenced by established cardiovascular risk factors is necessary to guide the use of cardiac troponin in cardiovascular risk estimation systems in women and men.

Using the longitudinal Whitehall II cohort study, we conducted a comprehensive sex-specific analysis of cardiac troponin trajectories over the life course, evaluating their determinants and relationship with cardiovascular outcomes in the general population.

## Methods

### Study Population

The Whitehall II study is an ongoing longitudinal observational cohort study of 10 308 British civil servants (6895 men and 3413 women) aged between 35–55 years old when first recruited in 1985.^15^ Follow-up has continued over 13 phases, with the most recent assessment completed in 2019. Stored samples were available for cardiac troponin testing from participants assessed on 3 occasions in 1997 to 1999, 2007 to 2009, and 2012 to 2013. We included all participants who had at least 1 measure of cardiac troponin and considered each participant’s first cardiac troponin measurement as baseline. The study was approved by the University College London Hospital Committee on the Ethics of Human Research (reference 85/0938), and the study was conducted according to the Declaration of Helsinki.

### Clinical Characteristics

For each participant we collected clinical characteristics at baseline and during follow-up. We collected data for the following clinical characteristics: age, sex, ethnic origin (White/other than White), diabetes mellitus, systolic blood pressure, total cholesterol concentration, high-density lipoprotein concentration, low-density lipoprotein concentration, smoking status (never/former/current), BMI, and medication prescriptions (lipid modifying medication/antihypertensive medication/angiotensin-converting enzyme inhibitors inhibitors/antiplatelets/ betablockers). In addition, we collected female-specific data on the menopausal transition (ie, age at which periods had stopped) and the use of female sex-hormone medication (oral contraceptives and hormone replacement therapy).

### Cardiac Troponin Measurements

Blood samples for each phase were handled according to a standardized protocol. Fasting venous blood samples were collected, centrifuged, and serum was stored in aliquots at −80 °C until batch analysis was performed. Cardiac troponin I concentrations were measured using the Siemens Atellica Immunoassay High Sensitivity Troponin I assay (Siemens Healthineers, Erlangen, Germany). This assay has a limit of blank of 0.5 ng/L, limit of detection of 1.6 ng/L and a limit of quantitation of 2.5 ng/L.^16^ The sex-specific 99th percentile upper reference limits are 34 ng/L and 53 ng/L in women and men, respectively. For the analyses, cardiac troponin concentrations below the limit of blank of 0.5 ng/L were assigned a value at the limit of blank.

### Clinical Outcomes

Outcomes were collected throughout the study period until March 2019 using the National Health Service Central Registry.^17^ Nonfatal events were defined using the Hospital Episode Statistics database records up to March 2019 using the 9th and 10th revision International Classification of Diseases (ICD-9 and ICD-10) codes where listed in the primary and secondary position.^18^ The primary outcome was a composite of nonfatal myocardial infarction (MI), nonfatal stroke, or cardiovascular death. Secondary outcomes were nonfatal MI, nonfatal stroke, cardiovascular death, non-cardiovascular death, and all-cause death. Clinical outcomes were defined using the ICD-9 and ICD-10 codes: cardiovascular death (ICD 9: 340–459 or ICD-10: I00–I99), nonfatal MI (ICD-9: 410 or ICD-10: I21) and nonfatal stroke (ICD-9: 430, 431, 434, 436 or ICD-10 codes: I60, I61, I63, I64), and non-cardiovascular death (all other ICD codes).

### Statistical Analysis

Continuous variables are presented as mean, standard deviation, or median, 25th to 75th percentile, as appropriate. Categorical variables are presented as absolute number (%).

We evaluated the temporal pattern of cardiac troponin in women and men over the middle-to-late adulthood life course using linear mixed-effects modeling. The distribution of cardiac troponin was skewed, and to achieve normal distribution we applied log_2_ transformation. Age was used as timescale and was entered as fixed effect and random effect in the model. Natural cubic splines in combination with likelihood ratio tests were used to assess non-linear associations. Average cardiac troponin values were estimated over a range of the 0.5th to the 99.5th percentile of age using the final linear mixed-effects model. An interaction term for sex and age was used to estimate the average cardiac troponin trajectories in women and men separately. Univariable and multivariable sex-specific linear mixed-effects models were used to evaluate the association of ethnicity and cardiovascular risk factors determined at baseline and during follow-up, including diabetes status, systolic blood pressure, total cholesterol, high-density lipoprotein, low-density lipoprotein, smoking status, and BMI with repeated measures of cardiac troponin. Repeated measures of the cardiovascular risk factors, assessed at time of cardiac troponin sampling, were entered in the models as fixed effects while repeated cardiac troponin measures were used as outcome. An interaction term for sex and the risk factor of interest was used to estimate associations with cardiac troponin in women and men separately.

Subsequently, we evaluated the sex-specific association of baseline cardiac troponin level and the rate of change during follow-up with the primary outcome. We estimated the sex-specific baseline cardiac troponin level and sex-specific slope parameter for each individual using linear mixed-effects modeling. Based on the results, we classified individuals into the following 4 groups: Group 1 = baseline level < median and change < median; Group 2 = baseline level < median and change ≥ median; Group 3 = baseline level ≥ median and change < median; and Group 4 = baseline level ≥ median and change ≥ median. The estimated median baseline concentration of cardiac troponin was 2.4 ng/L in women and 3.8 ng/L in men, and the median change in cardiac troponin concentration was a 4.4% increase per year in women and a 3.5% increase per year in men. The Kaplan-Meier method was applied to estimate the cumulative incidence of the primary outcome during follow-up, whereas log-rank test was used for between group comparisons. Non-cardiovascular death was considered as competing risk. Additionally, we applied sex-specific multistate joint modeling to evaluate the association between individual cardiac troponin trajectories and cardiovascular events in women and men separately. Joint modeling combines a linear mixed-effects model to describe the trajectory of a predictor with a time-to-event relative risk model to relate the estimated temporal pattern of a predictor with the hazard of the outcome of interest. In the context of repeated measurements, we not only studied the predictive value of cardiac troponin levels, but we also studied the predictive value of the slope of longitudinal trajectory (rate of change). Further details on the joint model application can be found in the Supplemental Material.^19^ Regarding the joint model, we adjusted for sex and age in the linear mixed-effects models, whereas unadjusted and adjusted time-to-event models were explored. Adjustment factors included age, diabetes, total cholesterol levels, high-density lipoprotein levels, low-density lipoprotein levels, systolic blood pressure, and smoking status at baseline. Non-cardiovascular death was included in the multistate joint model as a competing risk. The results are presented as hazard ratios (HRs) and 95% confidence intervals per 2-fold difference in cardiac troponin levels and slope (change in cardiac troponin concentration per 5 years).

In secondary analyses, we assessed the relationship of cardiac troponin with the cumulative incidence of nonfatal MI, nonfatal stroke, or death from any cause. Using multistate joint models, the relationship between sex-specific trajectories of cardiac troponin and all-cause death and non-cardiovascular death was evaluated. In addition, we explored the effect of menopause on the cardiac troponin trajectory in women using linear mixed-effects models. We anchored the timescale to the menopausal transition (ie, age at which periods stopped) to facilitate the analysis of repeated cardiac troponin in a time interval relative to menopause. For this analysis, we adjusted for the use of female sex-hormones at the time of cardiac troponin sampling. Women who reported that their period stopped as a result of hysterectomy (womb only), chemotherapy, or radiation therapy were excluded from this analysis. To evaluate whether cardiac troponin trajectories in women and men differ in those who have a subsequent MI or stroke, we conducted a sex-specific trajectory analysis where the primary outcome and individual components of the primary outcome were included as interaction terms in the linear mixed-effects models. Finally, we evaluated the sex-specific trajectories by ethnicity (White versus non-White).

Single imputation using mice was applied for the clinical characteristics with missing values using other individual’s clinical and outcome data. Statistical analysis was performed in R version 4.2.0 using packages “mice,” “nlme,” “cmprsk,” “survival,” and “JMbayes2.” Further details on R code for linear mixed-effects models and multistate joint models can be found in the Supplemental Material and the original R code for this study is available upon request.

## Results

### Clinical Characteristics of Study Population

The total study population comprised 7293 individuals of which 2142 (29.4%) were women and 5151 (70.6%) were men (58±7 and 57±7 years of age at baseline, respectively). At baseline, women had a higher prevalence of diabetes (5.0% versus 4.4%) and were more often current smokers (11.4% versus 8.5%) compared to men (Table 1). Moreover, women were prescribed more antihypertensive medication (19.7% versus 16.6%) but fewer antiplatelet agents (4.9% versus 8.1%) than men.

**Table 1.**
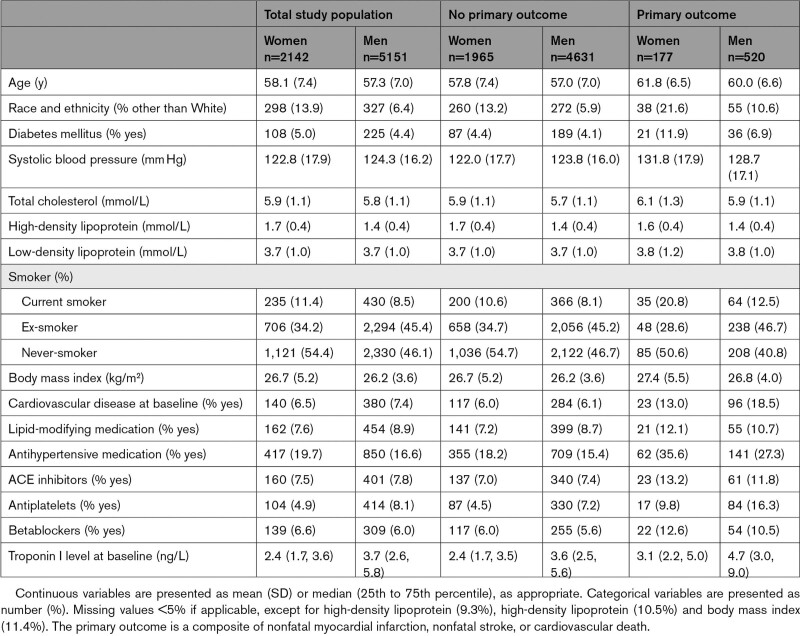
Baseline Characteristics of Study Population

A total of 1619 (75.6%) and 1090 (50.9%) women and 4199 (81.5%) and 2955 (57.4%) men had a second and third cardiac troponin measurement, respectively. Men were more likely to have detectable concentrations of cardiac troponin compared to women (Figure S1). There were 4847 (94.1%), 4156 (99.0%), and 2941 (99.5%) men, and 1677 (78.3%), 1538 (95.0%), and 1055 (96.8%) women, who had detectable cardiac troponin concentrations at their first, second, and third measurement, respectively. The time between first, second, and third cardiac troponin measurement was similar for women and men (Table S1).

### Sex-Specific Cardiac Troponin Trajectories Over the Life Course

**Figure 1.**
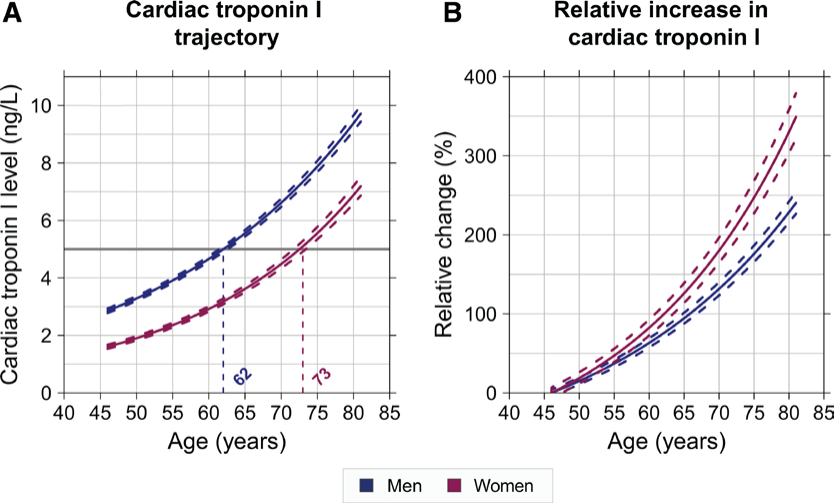
**Sex-specific cardiac troponin trajectories. A**, The sex-specific average trajectory of cardiac troponin over the middle to late adulthood life course. **B**, The sex-specific average relative increase in cardiac troponin from 46 years onwards. The solid red line depicts the average trajectory of cardiac troponin in women and the solid blue line depicts the average trajectory of cardiac troponin in men. The dashed lines represent the 95% CIs.

At baseline, median cardiac troponin concentrations were lower in women compared to men (2.4 [25th to 75th percentile, 1.7–3.6] ng/L versus 3.7 [25th to 75th percentile, 2.6–5.8] ng/L, *P*<0.001, Table 1). When evaluating the trajectories, both men and women showed an increase in cardiac troponin with advancing age, but concentrations remained lower in women compared to men over the entire middle-to-late adulthood life course (Figure 1A and Figure S2A). Despite their persistently lower cardiac troponin concentrations, women showed a larger relative increase from 46 years of age onwards (*P*_*interaction*_<0.001, Figure 1B and Figure S2B). Women reached equivalent cardiac troponin concentrations approximately a decade after men. For example, a cardiac troponin concentration of 5 ng/L, corresponds with an average age of 73 years and 62 years in women and men, respectively. No difference was observed in the average female-specific cardiac troponin trajectory before or after the menopausal transition (Figure S3). Women and men who experienced the primary outcome during follow-up showed a larger relative increase with advancing age compared to individuals who did not experience the primary outcome (*P*_*interaction*_<0.001, Figure S4). The relative increase of cardiac troponin in individuals with the primary outcome compared to those without the primary outcome was similar in women and men (*P*_*interaction*_=0.653). We did not find a significant interaction between the individual components of the primary outcome and sex-specific troponin trajectories (*P*_*interaction MI*_=0.572; *P*_*interaction stroke*_=0.349; *P*_*interaction CVD death*_=0.282). A similar pattern in sex-specific trajectories was found in White and non-White individuals (*P*_*interaction*_=0.907, Figure S5).

### Sex-Specific Longitudinal Relationship Between Risk Factors and Cardiac Troponin

**Figure 2.**
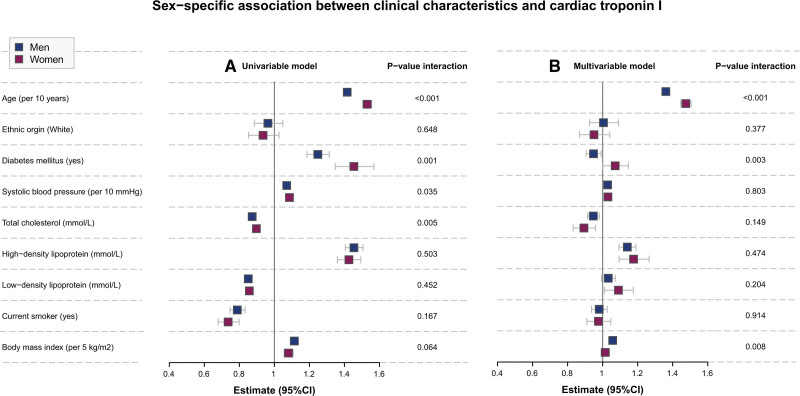
**Sex-specific association between clinical characteristics and cardiac troponin. A**, The sex-specific association between repeated measures of clinical characteristics and cardiac troponin in univariable linear mixed-effects analyses. **B**, The sex-specific association between repeated measures of clinical characteristics and cardiac troponin in a multivariable linear mixed-effects analysis. The multivariable model included all of the clinical characteristics evaluated.

We illustrate the longitudinal relationship between cardiovascular risk factors and cardiac troponin in women and men in Figure 2. In the multivariable model, age, diabetes, systolic blood pressure, total cholesterol, and high-density lipoprotein were significantly associated with cardiac troponin in both women and men. Increasing age was more strongly associated with an increase in cardiac troponin concentrations in women than men (*P*_*interaction*_<0.001, Figure 2B and Table S2). In contrast, BMI was more strongly associated with cardiac troponin in men than women (*P*_*interaction*_=0.008, Figure 2B and Table S2). Furthermore, diabetes is associated with increased cardiac troponin concentrations in women, while an inverse association was found in men (*P*_*interaction*_=0.003, Figure 2B and Table S2).

### Sex-Specific Association Between the Temporal Pattern of Cardiac Troponin and Primary Outcome

**Figure 3.**
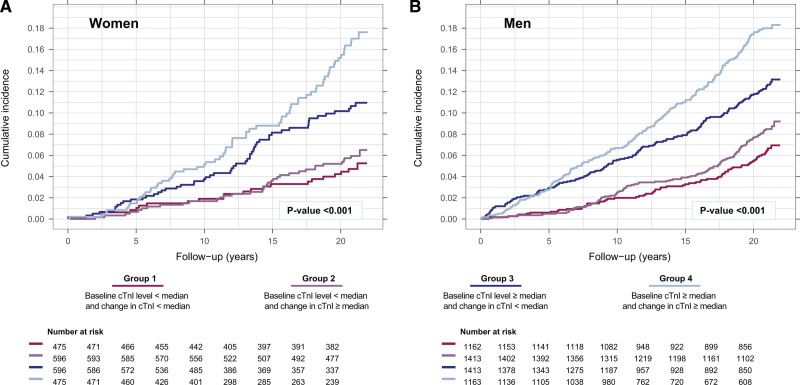
**Sex-specific association between cardiac troponin level and change over time and primary outcome.** Group 1 = baseline level < median and change < median; Group 2 = baseline level < median and change ≥ median; Group 3 = baseline level ≥ median and change < median; and Group 4 = baseline level ≥ median and change ≥ median. Log-rank test was used for between group comparisons. The estimated sex-specific median baseline level of cardiac troponin = 2.4 ng/L in women and 3.8 ng/L in men. The estimated sex-specific median change in cardiac troponin level equals a 4.4% increase per year in women and a 3.5% increase per year in men. The primary outcome is a composite of nonfatal myocardial infarction, nonfatal stroke, or cardiovascular death.

During a median follow-up of 20.9 (25th to 75th percentile, 15.8–21.3) years, the primary outcome occurred in 177 (8.3 %) women and 520 (10.1%) men (Table 1 and Table S3). We evaluated cardiac troponin concentrations at baseline combined with the change over time in relation to the cumulative incidence of the primary outcome. No differences were observed between women and men (Figure 3). Those with the lowest cardiac troponin level at baseline (Group 1 and Group 2) were at lowest risk, and those with the highest cardiac troponin level at baseline (Group 3 and Group 4) were at highest risk (Tables S4 and S5).

Using cardiac troponin trajectories, we showed that a 2-fold difference in cardiac troponin levels at any point in time during follow-up was numerically more strongly associated with the primary outcome in women (unadjusted HR, 1.57; 95% CI, 1.29–1.85; *P*<0.001) than in men (unadjusted HR, 1.38; 95% CI, 1.22–1.54; *P*<0.001; *P*_*interaction*_*=* 0.235; Table 2). The association persisted, although sex difference disappeared when we included known cardiovascular risk factors at baseline in the model (adjusted HR women, 1.34; 95% CI, 1.17–1.52; *P*<0.001 and adjusted HR men, 1.30; 95% CI, 1.21–1.40; *P*<0.001; *P*_*interaction*_*=* 0.752; Table 2). The slope in cardiac troponin was significantly associated with the primary outcome in women (adjusted HR women, 2.70; 95% CI, 1.01–7.33; *P=*0.049), but not in men (adjusted HR men, 1.31; 95% CI, 0.62–2.75; *P=*0.474; *P*_*interaction*_= 0.250).

**Table 2.**
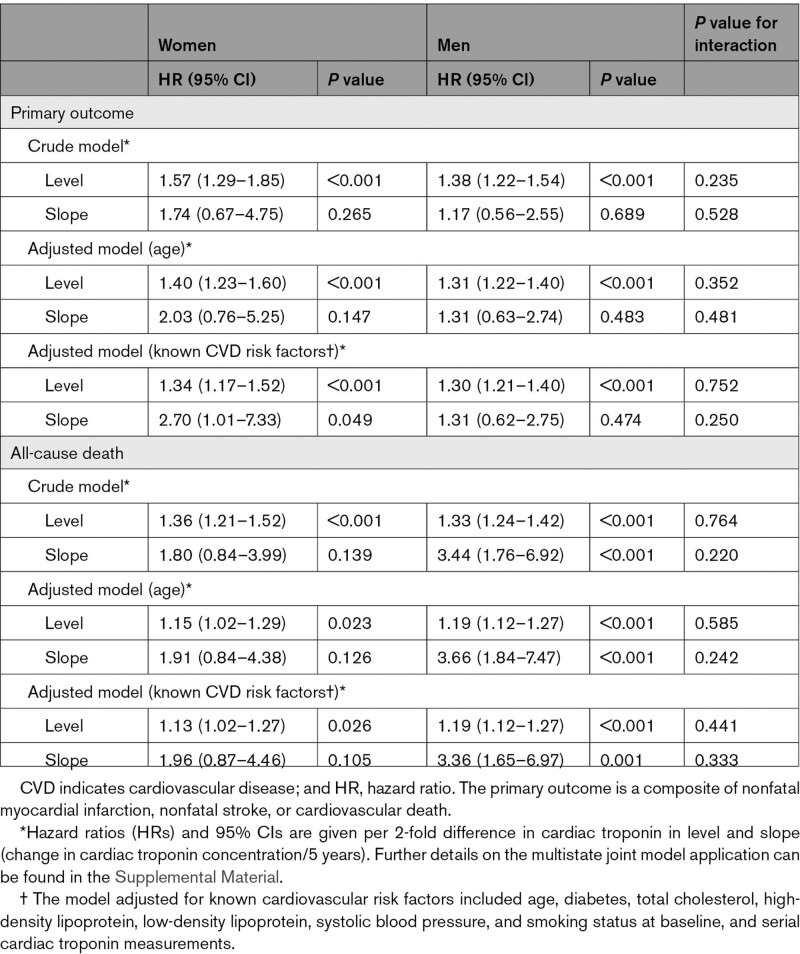
Sex-Specific Association Between Repeated Measurements of Cardiac Troponin I and Clinical Outcome

### Sex-Specific Association Between the Temporal Pattern of Cardiac Troponin and Secondary Outcomes

**Figure 4.**
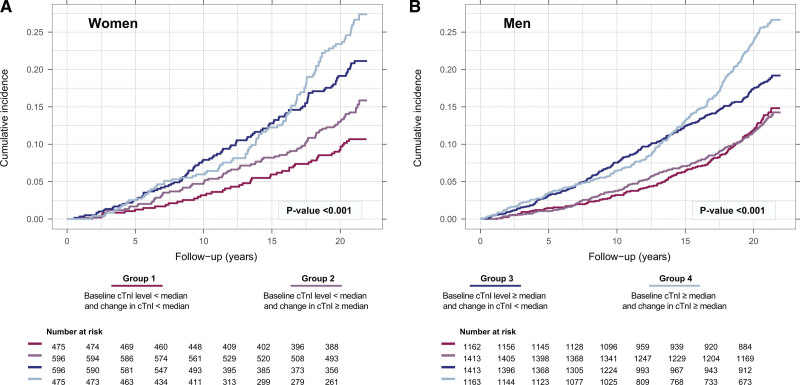
**Sex-specific association between cardiac troponin level and change over time and death from any cause.** Group 1 = baseline level < median and change < median; Group 2 = baseline level < median and change ≥ median; Group 3 = baseline level ≥ median and change < median; and Group 4 = baseline level ≥ median and change ≥ median. Log-rank test was used for between group comparisons. The estimated sex-specific median baseline level of cardiac troponin = 2.4 ng/L in women and 3.8 ng/L in men. The estimated sex-specific median change in cardiac troponin level equals a 4.4% increase per year in women and a 3.5% increase per year in men.

The cumulative incidence of death from any cause was similar between women and men: 14.0% and 14.1%, respectively (Table S3). In line with our primary analysis, we observed no sex-related difference in the association between the temporal pattern of cardiac troponin and all-cause death (Figure 4). Those with the lowest cardiac troponin level at baseline (Group 1 and Group 2) were at lowest risk, and those with the highest cardiac troponin level at baseline (Group 3 and Group 4) were at highest risk in both women and men. Similar observations were found for nonfatal MI, nonfatal stroke, cardiovascular death, and non-cardiovascular death (Figure S6 through S9).

We observed a 2-fold difference in cardiac troponin levels at any point in time during follow-up in those subsequently dying from any cause for both women (adjusted HR, 1.13; 95% CI, 1.02–1.27; *P*=0.026; Table 2) and men (adjusted HR, 1.19; 95% CI, 1.12–1.27; *P*<0.001; *P*_*interaction*_=0.441), independent of known cardiovascular risk factors. The slope of cardiac troponin was significantly associated with all-cause death in men (adjusted HR, 3.36; 95% CI, 1.65–6.97; *P*=0.001), but not in women (adjusted HR, 1.96; 95% CI, 0.87–4.46; *P*=0.105; *P*_*interaction*_=0.333). In contrast, no significant association between the temporal pattern of cardiac troponin and non-cardiovascular death was observed in the adjusted models (Table S6).

## Discussion

We conducted a comprehensive sex-specific analysis of the trajectories of cardiac troponin I over the life course in middle-to-late adulthood in the general population. Our study has 3 main findings. First, cardiac troponin concentrations in women lag around a decade behind men, but women exhibit a steeper trajectory with advancing age. Second, apart from age, we observed a significant interaction between sex and diabetes, and sex and BMI on the trajectory of cardiac troponin, suggesting that the impact of these risk factors on the development of subclinical cardiovascular disease may differ between women and men. Finally, the temporal pattern of cardiac troponin was more strongly related to cardiovascular events in women than men, although the association was attenuated after adjustment for cardiovascular risk factors. Our findings highlight the need for sex-specific approaches when cardiac troponin testing is applied in cardiovascular risk prediction.

In line with previous studies, cardiac troponin concentrations were persistently lower in women than men over the middle-to-late adulthood life course.^11–14^ We extend current knowledge by showing that women reach similar cardiac troponin concentrations approximately a decade after men, indicating that the development of coronary artery disease or other heart conditions may be delayed in women. In addition, we observed that women exhibit a greater relative increase in cardiac troponin with advancing age than men. Cardiovascular aging is a complex, multifactorial process, and sex differences arise from intrinsic biological variation as well as sex-specific changes in the myocardium and vasculature that manifest with aging.^20,21^ Although our exploratory analysis indicates that type of cardiovascular event does not appear to play a major role in the observed sex differences in troponin trajectories, differences in the prevalence of specific cardiovascular conditions, such as hypertension and atherosclerosis, in women and men may have contributed to a divergent distribution of cardiac troponin over time. Furthermore, it has been speculated that sex hormones may play a role in the divergent cardiac troponin concentrations between women and men.^22^ Estrogens seem to have a cardioprotective effect in premenopausal women, either directly or indirectly, which diminishes after menopause.^23–25^ Moreover, the testosterone to estradiol ratio, rather than the individual sex hormones, has been previously associated with increased risk of cardiovascular events in men.^26^ Our explorative analysis suggests no difference in the cardiac troponin trajectory of women pre- and post-menopause, but the role of sex-hormones should be elucidated in further longitudinal studies enrolling women in the decades prior to the menopause.

Apart from age, we identified that the longitudinal relationship between cardiac troponin and diabetes and BMI was modified by sex. A strong association between diabetes and cardiac troponin was observed in women, while an inverse association was observed in men. These findings align with the accumulating evidence suggesting that diabetes confers a higher cardiovascular risk in women than it does in men, independent of other risk factors.^27,28^ The diabetes-related increased risk of cardiovascular disease in women as compared to men is likely to be multifactorial.^29^ This may be due to the combination of a more rapid progression in cardiovascular and metabolic risk factor profile, delayed diagnosis and less intensive treatment of diabetes, more involvement of inflammatory factors, higher susceptibility to oxidative stress, and genetic or hormonal factors.^29^ The sex-related difference in the association between BMI and cardiac troponin may be attributed to different traits of adipose tissue. Namely, adipose tissue in women typically represents subcutaneous adipose tissue, whereas in men it represents visceral/abdominal adipose tissue.^30^ Subcutaneous adipose tissue exerts protective effects, whereas visceral/abdominal adipose tissue induces harmful metabolic alterations and increases the risk of cardiovascular disease.^30^

Previous studies have demonstrated that serial cardiac troponin testing is a promising tool for cardiovascular risk estimation,^8–10^ but whether a sex-specific approach should be considered when serial testing is applied has not yet been elucidated. The sex-specific association between cardiac troponin measured at a single timepoint and future cardiovascular risk is well established.^6,31–38^ We found in our study that the temporal pattern of cardiac troponin was independently associated with future cardiovascular events. In line with previous studies that evaluated cardiac troponin at a single time point, we show no relationship between the temporal pattern of cardiac troponin and non-cardiovascular deaths which is an important consideration when using cardiac troponin for risk prediction. We previously demonstrated that a sex-specific approach is required for risk prediction when using a single cardiac troponin measurement, but also that the interaction with sex is mostly explained by the prevalence of cardiovascular risk factors and prior disease.^34^ Similarly, the level of cardiac troponin was more strongly related to cardiovascular events in women than men in the current study, and this divergence by sex disappeared after adjustment for known cardiovascular risk factors. Our study suggests that the rate of change of cardiac troponin may be more informative in women than in men as we only observed a significant relationship between rate of change in cardiac troponin and the primary outcome in women. The findings of the current study highlight the importance of a sex-specific approach when using high-sensitivity cardiac troponin testing for risk stratification and targeting preventative treatments of cardiovascular disease. Use of the same thresholds to guide risk of future cardiovascular events in women and men would not provide equivalent prediction and such thresholds would be challenging to apply in older populations or diabetic individuals. Ideally, cardiac troponin would be used as a continuous, longitudinal measure in a dynamic cardiovascular risk prediction tool that incorporates sex, age, and other clinical features, thereby eliminating the problem of under- or overestimation for other important subgroups apart from sex. Such a dynamic risk prediction tool could provide a more individualized approach for both women and men to improve outcomes and provide additional public health benefits. A dynamic measure of risk could encourage patients to remain on therapy or guide clinicians to escalate therapy where risk remains elevated. Further work is needed to evaluate whether including serial measures of lifestyle factors (eg, diet and physical activity), traditional cardiovascular risk factors (eg, blood pressure and cholesterol), and other biomarkers (eg, N-terminal pro-B-type natriuretic peptide and creatinine) incorporated in an interactive and dynamic clinical tool could improve cardiovascular risk prediction in both women and men.

Our study has several strengths. First, our study is the first that conducted a sex-specific analysis of cardiac troponin trajectories in a general population setting. So far, studies on the sex-specific associations of cardiac troponin in the general population have traditionally performed cross-sectional measurements only^13^ or have left a short time interval between just 2 repeated measurements.^8,39^ Second, the Whitehall II study includes extensive phenotyping of almost the entire cohort, which allowed us to evaluate the sex-specific association between repeated measures of clinical characteristics and cardiac troponin. Third, complete follow-up for over 20 years ensured we had sufficient cardiovascular events to evaluate prediction of the cardiac troponin trajectory in men and women separately.

Some limitations of our study need to be acknowledged. First, men were overrepresented in the Whitehall II study. Second, the Whitehall II study comprises a mostly White population and generalizing our findings to other ethnic groups should be done with caution. Although we conducted a separate analysis in White and non-White individuals and found a similar pattern, further research is required to evaluate the impact of different ethnicities on troponin trajectories in women and men over the life course. Third, we relied on Hospital Episode Statistics to identify outcome events, and whilst this will result in some misclassification this is likely to be similar in women and men. Finally, we have only evaluated 1 cardiac troponin assay which precludes extrapolation of our findings to other cardiac troponin I assays.

In conclusion, trajectories of cardiac troponin differ between women and men in the general population, with differing associations to conventional risk factors and cardiovascular outcomes. Our findings highlight the importance of a sex-specific risk approach when serial cardiac troponin testing is applied to cardiovascular risk prediction.

## Article Information

### Acknowledgments

The authors gratefully acknowledge the British Heart Foundation Cardiovascular Biomarker Laboratory, the University of Edinburgh for their expertise and assistance in this work, and the support of participants in the Whitehall II study.

M.d.B, A.A., N.L.M., and D.M.K. conceived the study and its design. M.d.B., A.A., N.L.M., and D.M.K. had access to the data and performed the analysis. M.d.B., A.A., E.J.B., N.L.M., and D.M.K. interpreted the data and drafted the manuscript. All authors revised the manuscript critically for important intellectual content and provided their final approval of the version to be published. All authors are accountable for the work.

### Sources of Funding

The Whitehall II Study has been supported by grants from the British Medical Research Council; British Economic and Social Research Council; British Heart Foundation (RG/16/11/32334); United Kingdom Health and Safety Executive; United Kingdom Department of Health; National Heart Lung and Blood Institute (HL36310), National Institutes of Health; National Institute on Aging R01AG056477, RF1AG062553), National Institutes of Health; Agency for Health Care Policy Research (HS06516); John D. and Catherine T. MacArthur Foundation Research Networks on Successful Midlife Development and Socio-Economic Status and Health; United Kingdom Stroke Association; and the United Kingdom Health and Safety Executive. The study was supported by an investigator-initiated study grant from the Siemens Healthineers to the University of Edinburgh. Dr de Bakker is supported by the Jaap Schouten Foundation. Dr Shah is supported by a British Heart Foundation Intermediate Clinical Research Fellowship (FS/19/17/34172). Dr Brunner’s research is supported by UKRI (ES/T014377/1). Dr Mills is supported by a Chair Award (CH/F/21/90010), a Programme Grant (RG/20/10/34966), and a Research Excellence Award (RE/18/5/34216) from the British Heart Foundation. Dr Kimenai is supported by Health Data Research UK which receives its funding from Health Data Research UK Ltd (HDR-5012), funded by the UK Medical Research Council, Engineering and Physical Sciences Research Council, Economic and Social Research Council, Department of Health and Social Care (England), Chief Scientist Office of the Scottish Government Health and Social Care Directorates, Health and Social Care Research and Development Division (Welsh Government), Public Health Agency (Northern Ireland), British Heart Foundation, and the Wellcome Trust. The funders had no role in the study design, statistical analysis, or decision to submit this work to be considered for publication.

### Disclosures

Dr Shah has received honoraria from Abbott Diagnostics. Dr Mills has acted as a consultant for Abbott Diagnostics, Siemens Healthineers, Roche, and LumiraDx. The other authors have no conflicts of interest.

### Supplemental Material

Tables S1–S6

Figures S1–S9

The principle of joint modelling

R code and educational material of linear mixed-effects models and multistate joint models

## Supplementary Material

## References

[1] Bergström G Persson M Adiels M Björnson E Bonander C Ahlström H Alfredsson J Angerås O Berglund G Blomberg A. Prevalence of subclinical coronary artery atherosclerosis in the general population. Circulation. 2021;144:916–929. doi: 10.1161/CIRCULATIONAHA.121.055340 34543072 10.1161/CIRCULATIONAHA.121.055340 PMC8448414

[2] Blankenberg S Salomaa V Makarova N Ojeda F Wild P Lackner KJ Jørgensen T Thorand B Peters A Nauck M; BiomarCaRE Investigators. Troponin I and cardiovascular risk prediction in the general population: the BiomarCaRE consortium. Eur Heart J. 2016;37:2428–2437. doi: 10.1093/eurheartj/ehw172 27174290 10.1093/eurheartj/ehw172 PMC4982535

[3] Welsh P Preiss D Hayward C Shah ASV McAllister D Briggs A Boachie C McConnachie A Padmanabhan S Welsh C. Cardiac Troponin T and Troponin I in the General Population. Circulation. 2019;139:2754–2764. doi: 10.1161/CIRCULATIONAHA.118.038529 31014085 10.1161/CIRCULATIONAHA.118.038529 PMC6571179

[4] Willeit P Welsh P Evans JDW Tschiderer L Boachie C Jukema JW Ford I Trompet S Stott DJ Kearney PM. High-sensitivity cardiac troponin concentration and risk of first-ever cardiovascular outcomes in 154,052 participants. J Am Coll Cardiol. 2017;70:558–568. doi: 10.1016/j.jacc.2017.05.062 28750699 10.1016/j.jacc.2017.05.062 PMC5527070

[5] Daniels LB Clopton P deFilippi CR Sanchez OA Bahrami H Lima JA Tracy RP Siscovick D Bertoni AG Greenland P. Serial measurement of N-terminal pro-B-type natriuretic peptide and cardiac troponin T for cardiovascular disease risk assessment in the Multi-Ethnic Study of Atherosclerosis (MESA). Am Heart J. 2015;170:1170–1183. doi: 10.1016/j.ahj.2015.09.010 26678639 10.1016/j.ahj.2015.09.010 PMC4684596

[6] Jia X Sun W Hoogeveen RC Nambi V Matsushita K Folsom AR Heiss G Couper DJ Solomon SD Boerwinkle E. High-sensitivity troponin I and incident coronary events, stroke, heart failure hospitalization, and mortality in the ARIC study. Circulation. 2019;139:2642–2653. doi: 10.1161/CIRCULATIONAHA.118.038772 31030544 10.1161/CIRCULATIONAHA.118.038772 PMC6546524

[7] de Lemos JA Drazner MH Omland T Ayers CR Khera A Rohatgi A Hashim I Berry JD Das SR Morrow DA. Association of troponin T detected with a highly sensitive assay and cardiac structure and mortality risk in the general population. JAMA. 2010;304:2503–2512. doi: 10.1001/jama.2010.1768 21139111 10.1001/jama.2010.1768 PMC5621378

[8] Lyngbakken MN Røsjø H Holmen OL Dalen H Hveem K Omland T. Temporal changes in cardiac troponin I are associated with risk of cardiovascular events in the general population: the Nord-Trøndelag Health study. Clin Chem. 2019;65:871–881. doi: 10.1373/clinchem.2018.301069 30996050 10.1373/clinchem.2018.301069

[9] Hughes MF Ojeda F Saarela O Jørgensen T Zeller T Palosaari T O’Doherty MG Borglykke A Kuulasmaa K Blankenberg S. Association of repeatedly measured high-sensitivity-assayed troponin I with cardiovascular disease events in a general population from the MORGAM/BiomarCaRE study. Clin Chem. 2017;63:334–342. doi: 10.1373/clinchem.2016.261172 28062627 10.1373/clinchem.2016.261172

[10] Ford I Shah AS Zhang R McAllister DA Strachan FE Caslake M Newby DE Packard CJ Mills NL. High-sensitivity cardiac troponin, statin therapy, and risk of coronary heart disease. J Am Coll Cardiol. 2016;68:2719–2728. doi: 10.1016/j.jacc.2016.10.020 28007133 10.1016/j.jacc.2016.10.020 PMC5176330

[11] Mingels A Jacobs L Michielsen E Swaanenburg J Wodzig W van Dieijen-Visser M. Reference population and marathon runner sera assessed by highly sensitive cardiac troponin T and commercial cardiac troponin T and I assays. Clin Chem. 2009;55:101–108. doi: 10.1373/clinchem.2008.106427 18988757 10.1373/clinchem.2008.106427

[12] Kimenai DM Henry RM van der Kallen CJ Dagnelie PC Schram MT Stehouwer CD van Suijlen JD Niens M Bekers O Sep SJ. Direct comparison of clinical decision limits for cardiac troponin T and I. Heart. 2016;102:610–616. doi: 10.1136/heartjnl-2015-308917 26794233 10.1136/heartjnl-2015-308917

[13] Kimenai DM Janssen E Eggers KM Lindahl B den Ruijter HM Bekers O Appelman Y Meex SJR. Sex-specific versus overall clinical decision limits for cardiac troponin I and T for the diagnosis of acute myocardial infarction: a systematic review. Clin Chem. 2018;64:1034–1043. doi: 10.1373/clinchem.2018.286781 29844245 10.1373/clinchem.2018.286781

[14] Apple FS Sandoval Y Jaffe AS Ordonez-Llanos J; IFCC Task Force on Clinical Applications of Cardiac Bio-Markers. Cardiac troponin assays: guide to understanding analytical characteristics and their impact on clinical care. Clin Chem. 2017;63:73–81. doi: 10.1373/clinchem.2016.255109 28062612 10.1373/clinchem.2016.255109

[15] Marmot M Brunner E. Cohort profile: the Whitehall II study. Int J Epidemiol. 2005;34:251–256. doi: 10.1093/ije/dyh372 15576467 10.1093/ije/dyh372

[16] Chapman AR Fujisawa T Lee KK Andrews JP Anand A Sandeman D Ferry AV Stewart S Marshall L Strachan FE. Novel high-sensitivity cardiac troponin I assay in patients with suspected acute coronary syndrome. Heart. 2019;105:616–622. doi: 10.1136/heartjnl-2018-314093 30442743 10.1136/heartjnl-2018-314093 PMC6580754

[17] Hinnouho GM Czernichow S Dugravot A Nabi H Brunner EJ Kivimaki M Singh-Manoux A. Metabolically healthy obesity and the risk of cardiovascular disease and type 2 diabetes: the Whitehall II cohort study. Eur Heart J. 2015;36:551–559. doi: 10.1093/eurheartj/ehu123 24670711 10.1093/eurheartj/ehu123 PMC4344958

[18] Kivimäki M Batty GD Singh-Manoux A Britton A Brunner EJ Shipley MJ. Validity of cardiovascular disease event ascertainment using linkage to UK hospital records. Epidemiology. 2017;28:735–739. doi: 10.1097/EDE.0000000000000688 28570383 10.1097/EDE.0000000000000688 PMC5540351

[19] Rizopoulos D. Joint Models for Longitudinal and Time-to-Event Data, with Applications in R. Boca Raton: Chapman & Hall/CRC; 2012.

[20] Merz AA Cheng S. Sex differences in cardiovascular ageing. Heart. 2016;102:825–831. doi: 10.1136/heartjnl-2015-308769 26917537 10.1136/heartjnl-2015-308769 PMC5993677

[21] Ji H Kwan AC Chen MT Ouyang D Ebinger JE Bell SP Niiranen TJ Bello NA Cheng S. Sex differences in myocardial and vascular aging. Circ Res. 2022;130:566–577. doi: 10.1161/CIRCRESAHA.121.319902 35175845 10.1161/CIRCRESAHA.121.319902 PMC8863105

[22] Subramanya V Zhao D Ouyang P Lima JA Vaidya D Ndumele CE Bluemke DA Shah SJ Guallar E Nwabuo CC. Sex hormone levels and change in left ventricular structure among men and post-menopausal women: The Multi-Ethnic Study of Atherosclerosis (MESA). Maturitas. 2018;108:37–44. doi: 10.1016/j.maturitas.2017.11.006 29290213 10.1016/j.maturitas.2017.11.006 PMC5752123

[23] Piro M Della Bona R Abbate A Biasucci LM Crea F. Sex-related differences in myocardial remodeling. J Am Coll Cardiol. 2010;55:1057–1065. doi: 10.1016/j.jacc.2009.09.065 20223363 10.1016/j.jacc.2009.09.065

[24] Westerman S Wenger NK. Women and heart disease, the underrecognized burden: sex differences, biases, and unmet clinical and research challenges. Clin Sci (Lond). 2016;130:551–563. doi: 10.1042/CS20150586 26957643 10.1042/CS20150586

[25] Iorga A Cunningham CM Moazeni S Ruffenach G Umar S Eghbali M. The protective role of estrogen and estrogen receptors in cardiovascular disease and the controversial use of estrogen therapy. Biol Sex Differ. 2017;8:33. doi: 10.1186/s13293-017-0152-8 29065927 10.1186/s13293-017-0152-8 PMC5655818

[26] van Koeverden ID de Bakker M Haitjema S van der Laan SW de Vries J-PPM Hoefer IE de Borst GJ Pasterkamp G den Ruijter HM. Testosterone to oestradiol ratio reflects systemic and plaque inflammation and predicts future cardiovascular events in men with severe atherosclerosis. Cardiovasc Res. 2018;115:453–462. doi: 10.1093/cvr/cvy188 10.1093/cvr/cvy188 30052805

[27] Madonna R Balistreri CR De Rosa S Muscoli S Selvaggio S Selvaggio G Ferdinandy P De Caterina R. Impact of sex differences and diabetes on coronary atherosclerosis and ischemic heart disease. J Clin Med. 2019;8:98. doi: 10.3390/jcm8010098 30654523 10.3390/jcm8010098 PMC6351940

[28] Malmborg M Schmiegelow MDS Nørgaard CH Munch A Gerds T Schou M Kistorp C Torp-Pedersen C Hlatky MA Gislason G. Does type 2 diabetes confer higher relative rates of cardiovascular events in women compared with men? Eur Heart J. 2020;41:1346–1353. doi: 10.1093/eurheartj/ehz913 31860067 10.1093/eurheartj/ehz913 PMC7109603

[29] Regensteiner JG Golden S Huebschmann AG Barrett-Connor E Chang AY Chyun D Fox CS Kim C Mehta N Reckelhoff JF; American Heart Association Diabetes Committee of the Council on Lifestyle and Cardiometabolic Health, Council on Epidemiology and Prevention, Council on Functional Genomics and Translational Biology, and Council on Hypertension. Sex differences in the cardiovascular consequences of diabetes mellitus: a scientific statement from the American Heart Association. Circulation. 2015;132:2424–2447. doi: 10.1161/CIR.0000000000000343 26644329 10.1161/CIR.0000000000000343

[30] Lumish HS O’Reilly M Reilly MP. Sex differences in genomic drivers of adipose distribution and related cardiometabolic disorders. Arterioscler Thromb Vasc Biol. 2020;40:45–60. doi: 10.1161/ATVBAHA.119.313154 31747800 10.1161/ATVBAHA.119.313154

[31] Dallmeier D Denkinger M Peter R Rapp K Jaffe AS Koenig W Rothenbacher D; ActiFE Study Group. Sex-specific associations of established and emerging cardiac biomarkers with all-cause mortality in older adults: the ActiFE study. Clin Chem. 2015;61:389–399. doi: 10.1373/clinchem.2014.230839 25501933 10.1373/clinchem.2014.230839

[32] Eggers KM Johnston N Lind L Venge P Lindahl B. Cardiac troponin I levels in an elderly population from the community — the implications of sex. Clin Biochem. 2015;48:751–756. doi: 10.1016/j.clinbiochem.2015.04.013 25916815 10.1016/j.clinbiochem.2015.04.013

[33] Thorsteinsdottir I Aspelund T Gudmundsson E Eiriksdottir G Harris TB Launer LJ Gudnason V Venge P. High-sensitivity cardiac troponin I is a strong predictor of cardiovascular events and mortality in the AGES-Reykjavik Community-Based Cohort of Older Individuals. Clin Chem. 2016;62:623–630. doi: 10.1373/clinchem.2015.250811 26936931 10.1373/clinchem.2015.250811 PMC5943042

[34] Kimenai DM Shah ASV McAllister DA Lee KK Tsanas A Meex SJR Porteous DJ Hayward C Campbell A Sattar N. Sex differences in cardiac troponin I and T and the prediction of cardiovascular events in the general population. Clin Chem. 2021;67:1351–1360. doi: 10.1093/clinchem/hvab109 34240125 10.1093/clinchem/hvab109 PMC8486023

[35] Omland T de Lemos JA Holmen OL Dalen H Benth J Nygård S Hveem K Røsjø H. Impact of sex on the prognostic value of high-sensitivity cardiac troponin I in the general population: the HUNT study. Clin Chem. 2015;61:646–656. doi: 10.1373/clinchem.2014.234369 25695851 10.1373/clinchem.2014.234369

[36] Saunders JT Nambi V de Lemos JA Chambless LE Virani SS Boerwinkle E Hoogeveen RC Liu X Astor BC Mosley TH. Cardiac troponin T measured by a highly sensitive assay predicts coronary heart disease, heart failure, and mortality in the Atherosclerosis Risk in Communities Study. Circulation. 2011;123:1367–1376. doi: 10.1161/CIRCULATIONAHA.110.005264 21422391 10.1161/CIRCULATIONAHA.110.005264 PMC3072024

[37] Lyngbakken MN Røsjø H Holmen OL Nygård S Dalen H Hveem K Omland T. Gender, high-sensitivity troponin I, and the risk of cardiovascular events (from the Nord-Trøndelag Health study). Am J Cardiol. 2016;118:816–821. doi: 10.1016/j.amjcard.2016.06.043 27453509 10.1016/j.amjcard.2016.06.043

[38] Eggers KM Johnston N James S Lindahl B Venge P. Cardiac troponin I levels in patients with non-ST-elevation acute coronary syndrome-the importance of gender. Am Heart J. 2014;168:317–324.e1. doi: 10.1016/j.ahj.2014.06.006 25173543 10.1016/j.ahj.2014.06.006

[39] Eggers KM Lind L Venge P Lindahl B. Factors influencing the 99th percentile of cardiac troponin I evaluated in community-dwelling individuals at 70 and 75 years of age. Clin Chem. 2013;59:1068–1073. doi: 10.1373/clinchem.2012.196634 23462029 10.1373/clinchem.2012.196634

