# Supplementary material for: Sex Differences in Cardiac Troponin Trajectories Over the Life Course
Source: Circulation. 2023 Apr 28;147(24):1798–808. doi: 10.1161/CIRCULATIONAHA.123.064386 (PMC10249606; doi:10.1161/CIRCULATIONAHA.123.064386)
Supplement: Supplementary file 1 [file cir-147-1798-s001.pdf]

SUPPLEMENTAL MATERIAL

Sex differences in cardiac troponin trajectories over the life course

Running title: Sex differences in cardiac troponin trajectories

Marie de Bakker, MSc[1], Atul Anand, MD, PhD[2], Martin Shipley, MSc[3],

Takeshi Fujisawa, PhD[2], Anoop SV Shah, MD, PhD[4], Isabella Kardys, MD, PhD[1],

Eric Boersma, PhD[1], Eric J Brunner, PhD[3], Nicholas L Mills, MD, PhD[2,5],

Dorien M Kimenai, PhD[2]

[1] *Department of Cardiology, Erasmus MC Cardiovascular Institute, University Medical Center Rotterdam, Rotterdam, the Netherlands*
[2] *BHF Centre for Cardiovascular Science, University of Edinburgh, Edinburgh, United Kingdom*
[3] *Department of Epidemiology and Public Health, University College London, London, United Kingdom*
[4] *Department of non-communicable disease, London School of Hygiene and Tropical Medicine, London, United Kingdom*
[5] *Usher Institute, University of Edinburgh, Edinburgh, United Kingdom*

Supplemental Table 1. Time between first, second, and third cardiac troponin measurement

	Women	Men
Time between first and second measurement (years)	10.3 [9.9 to 10.6]	10.4 [10.0 to 10.8]
Time between second and third measurement (years)	4.1 [4.0 to 4.3]	4.1 [4.0 to 4.2]

Data are presented as median [25th to 75th percentile].

Supplemental Table 2. Sex-specific association between the repeatedly assessed clinical characteristics and longitudinal cardiac troponin I

	Women		Men			Women		Men		
	**Univariable estimate (95% CI)**	**P-value**	**Univariable estimate (95% CI)**	**P-value**	**P-value for interaction**	**Multivariable estimate (95% CI)**	**P-value**	**Multivariable estimate (95% CI)**	**P-value**	**P-value for interaction**
Age (per 10 years)	1.53 (1.50 to 1.56)	**<0.001**	1.42 (1.40 to 1.43)	**<0.001**	**<0.001**	1.47 (1.45 to 1.50)	**<0.001**	1.36 (1.34 to 1.38)	**<0.001**	**<0.001**
Ethnic origin (White)	0.94 (0.85 to 1.03)	0.158	0.96 (0.88 to 1.05)	0.385	0.648	0.95 (0.87 to 1.04)	0.275	1.00 (0.93 to 1.09)	0.912	0.377
Diabetes mellitus (yes)	1.45 (1.35 to 1.57)	**<0.001**	1.25 (1.19 to 1.31)	**<0.001**	**0.001**	1.07 (1.00 to 1.15)	**0.044**	0.95 (0.91 to 0.99)	**0.019**	**0.003**
Systolic blood pressure (per 10 mmHg)	1.09 (1.07 to 1.10)	**<0.001**	1.07 (1.06 to 1.08)	**<0.001**	**0.035**	1.03 (1.02 to 1.04)	**<0.001**	1.03 (1.02 to 1.04)	**<0.001**	0.803
Total cholesterol (mmol/L)	0.90 (0.88 to 0.91)	**<0.001**	0.87 (0.86 to 0.88)	**<0.001**	**0.005**	0.89 (0.83 to 0.96)	**0.002**	0.95 (0.91 to 0.98)	**0.002**	0.149
High-density lipoprotein (mmol/L)	1.43 (1.36 to 1.49)	**<0.001**	1.45 (1.40 to 1.51)	**<0.001**	0.503	1.18 (1.09 to 1.27)	**<0.001**	1.14 (1.09 to 1.19)	**<0.001**	0.474
Low-density lipoprotein (mmol/L)	0.86 (0.84 to 0.87)	**<0.001**	0.85 (0.84 to 0.86)	**<0.001**	0.452	1.09 (1.01 to 1.18)	**0.025**	1.03 (0.99 to 1.07)	0.108	0.204
Current smoker (yes)	0.74 (0.68 to 0.80)	**<0.001**	0.79 (0.75 to 0.83)	**<0.001**	0.167	0.98 (0.91 to 1.05)	0.496	0.98 (0.94 to 1.03)	0.396	0.914
Body mass index (per 5 kg/m2)	1.08 (1.05 to 1.11)	**<0.001**	1.11 (1.09 to 1.14)	**<0.001**	0.064	1.01 (0.99 to 1.04)	0.216	1.06 (1.04 to 1.08)	**<0.001**	**0.008**

Abbreviations: CI, confidence interval.

Supplemental Table 3. Sex-specific cumulative incidence of cardiovascular events at 1, 5, 10 and 20 years

	Women	Men
**Primary outcome**		
At 1 year	3 (0.1%)	23 (0.4%)
At 5 years	26 (1.2%)	87 (1.7%)
At 10 years	65 (3.1%)	213 (4.2%)
At 20 years	159 (8.3%)	482 (10.3%)
**Non-fatal myocardial infarction**		
At 1 year	1 (0.0%)	14 (0.3%)
At 5 years	7 (0.3%)	40 (0.8%)
At 10 years	24 (1.1%)	103 (2.0%)
At 20 years	52 (2.7%)	202 (4.3%)
**Non-fatal stroke**		
At 1 year	2 (0.1%)	5 (0.1%)
At 5 years	11 (0.5%)	23 (0.4%)
At 10 years	25 (1.2%)	67 (1.3%)
At 20 years	66 (3.5%)	186 (4.1%)
**Cardiovascular death**		
At 1 year	0 (0.0%)	5 (0.1%)
At 5 years	10 (0.5%)	31 (0.6%)
At 10 years	22 (1.0%)	67 (1.3%)
At 20 years	65 (3.0%)	174 (3.4%)
**Non-cardiovascular death**		
At 1 year	3 (0.1%)	7 (0.1%)
At 5 years	33 (1.5%)	86 (1.7%)
At 10 years	96 (4.5%)	202 (3.9%)
At 20 years	235 (11.0%)	551 (10.7%)
**Death from any cause**		
At 1 year	3 (0.1%)	12 (0.2%)
At 5 years	43 (2.0%)	43 (2.3%)
At 10 years	118 (5.5%)	269 (5.2%)
At 20 years	300 (14.0%)	725 (14.1%)

Supplemental Table 4. Clinical characteristics at baseline of female-specific cardiac troponin groups

	**Group 1*** *n = 475*	**Group 2*** *n = 596*	**Group 3*** *n = 596*	**Group 4*** *n = 475*
Age (years)	55.9 (6.9)	54.6 (5.3)	60.3 (8.0)	61.9 (6.4)
Ethnic origin (other than white) (%)	49 (10.3)	84 (14.1)	95 (16.0)	70 (14.8)
Diabetes mellitus (% yes)	20 (4.2)	19 (3.2)	38 (6.4)	31 (6.5)
Systolic blood pressure (mmHg)	119.0 (16.7)	118.3 (15.2)	126.9 (19.0)	127.1 (18.5)
Total cholesterol (mmol/L)	5.9 (1.1)	6.0 (1.1)	5.9 (1.1)	6.0 (1.1)
High-density lipoprotein (mmol/L)	1.7 (0.5)	1.7 (0.4)	1.7 (0.4)	1.7 (0.5)
Low-density lipoprotein (mmol/L)	3.7 (1.0)	3.8 (1.0)	3.6 (1.0)	3.8 (1.1)
Smoker (%)				
Current smoker	48 (10.4)	78 (13.5)	63 (11.0)	46 (10.2)
Ex-smoker	155 (33.6)	174 (30.2)	209 (36.5)	168 (37.3)
Never-smoker	258 (56.0)	325 (56.3)	301 (52.5)	237 (52.5)
Body mass index (kg/m2)	26.0 (4.9)	26.0 (4.9)	27.4 (5.3)	27.4 (5.5)
Lipid-modifying medication (% yes)	22 (4.7)	12 (2.0)	74 (12.4)	54 (11.5)
Antihypertensive medication (% yes)	66 (14.1)	55 (9.3)	166 (27.9)	130 (27.8)
ACE inhibitors (% yes)	23 (4.9)	14 (2.4)	69 (11.6)	54 (11.5)
Antiplatelets (% yes)	9 (1.9)	9 (1.5)	51 (8.6)	35 (7.5)
Betablockers (% yes)	22 (4.7)	15 (2.5)	57 (9.6)	45 (9.6)
Troponin I level at baseline (ng/L)	2.0 [1.6, 2.3]	1.5 [1.1, 1.8]	3.6 [3.0, 5.5]	3.6 [2.7, 5.9]

Abbreviations: ACE, angiotensin-converting enzyme.
Continuous variables are presented as mean (standard deviation) or median [25th to 75th percentile], as appropriate. Categorical variables are presented as number (%).
* Group 1 = baseline level < median and change < median; Group 2 = baseline level < median and change ≥ median; Group 3 = baseline level ≥ median and change < median; Group 4 = baseline level ≥ median and change ≥ median.

Supplemental Table 5. Clinical characteristics at baseline of male-specific cardiac troponin groups

	**Group 1***<br>*n = 1162*	**Group 2***<br>*n = 1413*	**Group 3***<br>*n = 1413*	**Group 4***<br>*n = 1163*
Age (years)	56.7 (7.0)	54.2 (5.3)	58.5 (7.5)	60.3 (6.5)
Ethnic origin (other than white) (%)	83 (7.1)	59 (4.2)	101 (7.2)	84 (7.2)
Diabetes mellitus (% yes)	46 (4.0)	48 (3.4)	66 (4.7)	65 (5.6)
Systolic blood pressure (mmHg)	122.3 (15.6)	121.4 (14.0)	127.2 (17.2)	126.5 (16.9)
Total cholesterol (mmol/L)	5.8 (1.0)	5.9 (1.0)	5.7 (1.2)	5.7 (1.1)
High-density lipoprotein (mmol/L)	1.4 (0.3)	1.4 (0.3)	1.4 (0.4)	1.4 (0.4)
Low-density lipoprotein (mmol/L)	3.8 (1.0)	3.8 (0.9)	3.6 (1.0)	3.6 (1.0)
Smoker (%)				
Current smoker	92 (8.0)	155 (11.1)	108 (7.8)	75 (6.6)
Ex-smoker	514 (44.9)	611 (43.9)	615 (44.6)	554 (48.6)
Never-smoker	538 (47.0)	625 (44.9)	655 (47.5)	512 (44.9)
Body mass index (kg/m2)	25.7 (3.5)	26.1 (3.5)	26.6 (3.9)	26.4 (3.5)
Lipid-modifying medication (% yes)	68 (5.9)	62 (4.4)	184 (13.1)	140 (12.1)
Antihypertensive medication (% yes)	134 (11.6)	122 (8.7)	302 (21.6)	292 (25.2)
ACE inhibitors (% yes)	56 (4.9)	44 (3.1)	151 (10.8)	150 (13.0)
Antiplatelets (% yes)	54 (4.7)	46 (3.3)	168 (12.0)	146 (12.6)
Betablockers (% yes)	62 (5.4)	47 (3.4)	114 (8.1)	86 (7.4)
Troponin I level at baseline (ng/L)	2.9 [2.4, 3.4]	2.3 [1.8, 2.8]	6.3 [4.9, 10.0]	5.1 [4.0, 8.1]

Abbreviations: ACE, angiotensin-converting enzyme.
Continuous variables are presented as mean (standard deviation) or median [25th to 75th percentile], as appropriate. Categorical variables are presented as number (%).
* Group 1 = baseline level < median and change < median; Group 2 = baseline level < median and change ≥ median; Group 3 = baseline level ≥ median and change < median; Group 4 = baseline level ≥ median and change ≥ median.

Supplemental Table 6. Sex-specific association between repeated measurements of cardiac troponin I and non-cardiovascular death

Non-cardiovascular death	Women HR (95% CI)	P-value	Men HR (95% CI)	P-value	P-value for interaction
**Crude model***					
Level	1.15 (1.00 to 1.31)	0.059	1.14 (1.04 to 1.25)	**0.004**	0.966
Slope	1.68 (0.66 to 4.27)	0.279	2.41 (1.03 to 5.40)	**0.043**	0.575
**Adjusted model (age) ***					
Level	0.96 (0.83 to 1.11)	0.613	1.01 (0.93 to 1.10)	0.824	0.580
Slope	1.91 (0.68 to 5.30)	0.217	2.43 (1.00 to 6.05)	0.051	0.730
**Adjusted model (known CVD**					
Level	0.96 (0.83 to 1.11)	0.607	1.01 (0.93 to 1.10)	0.775	0.560
Slope	1.96 (0.71 to 5.55)	0.197	2.37 (0.98 to 5.56)	0.056	0.782

Abbreviations: CI, confidence interval; HR, hazard ratio.
[*] Hazard ratios (HRs) and 95% confidence intervals (CIs) are given per two-fold difference in cardiac troponin in level and slope (change in cardiac troponin concentration/ 5 years). Further details on the multistate joint model application can be found in the Supplemental Material.
[†] The model adjusted for known cardiovascular risk factors included age, diabetes, total cholesterol, high-density lipoprotein, low-density lipoprotein, systolic blood pressure, and smoking status at baseline, and serial cardiac troponin measurements.

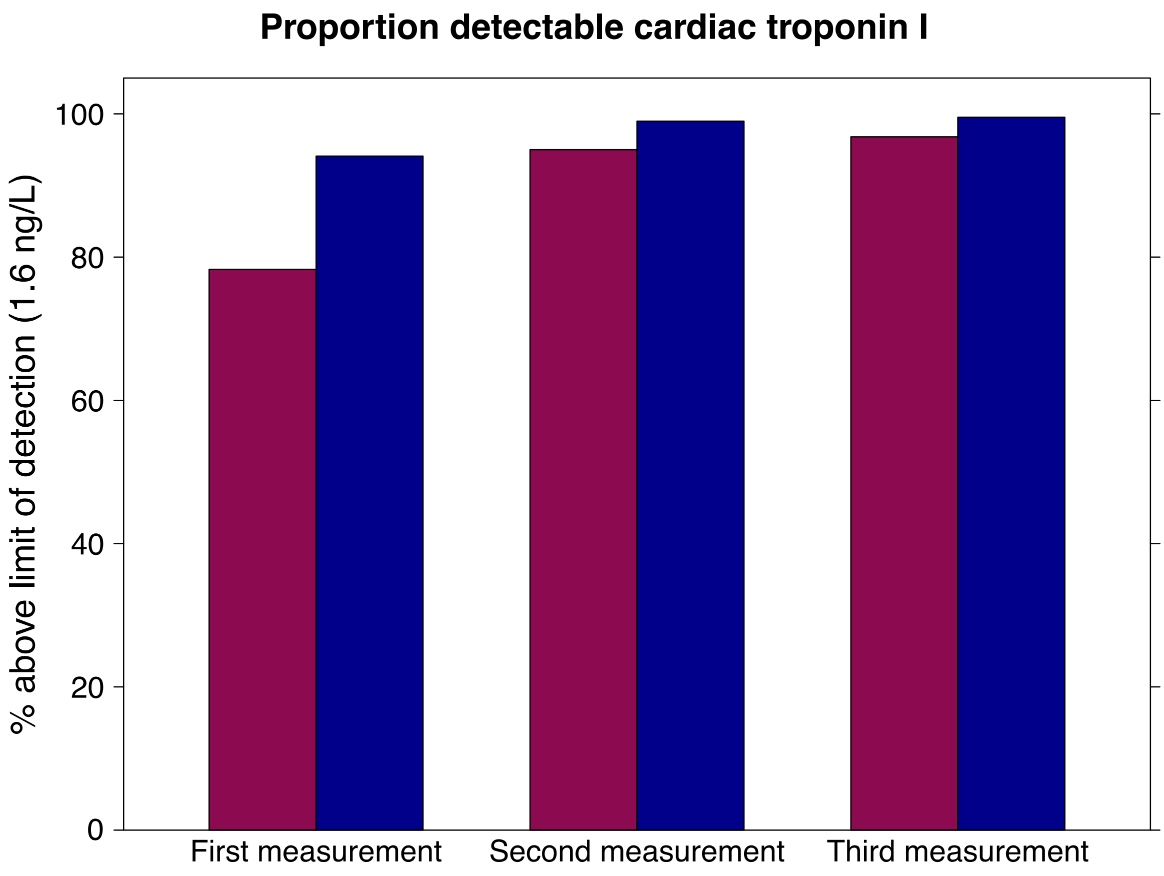

Supplemental Figure 1. Sex-specific proportion of detectable cardiac troponin I (≥1.6 ng/L) per measurement.

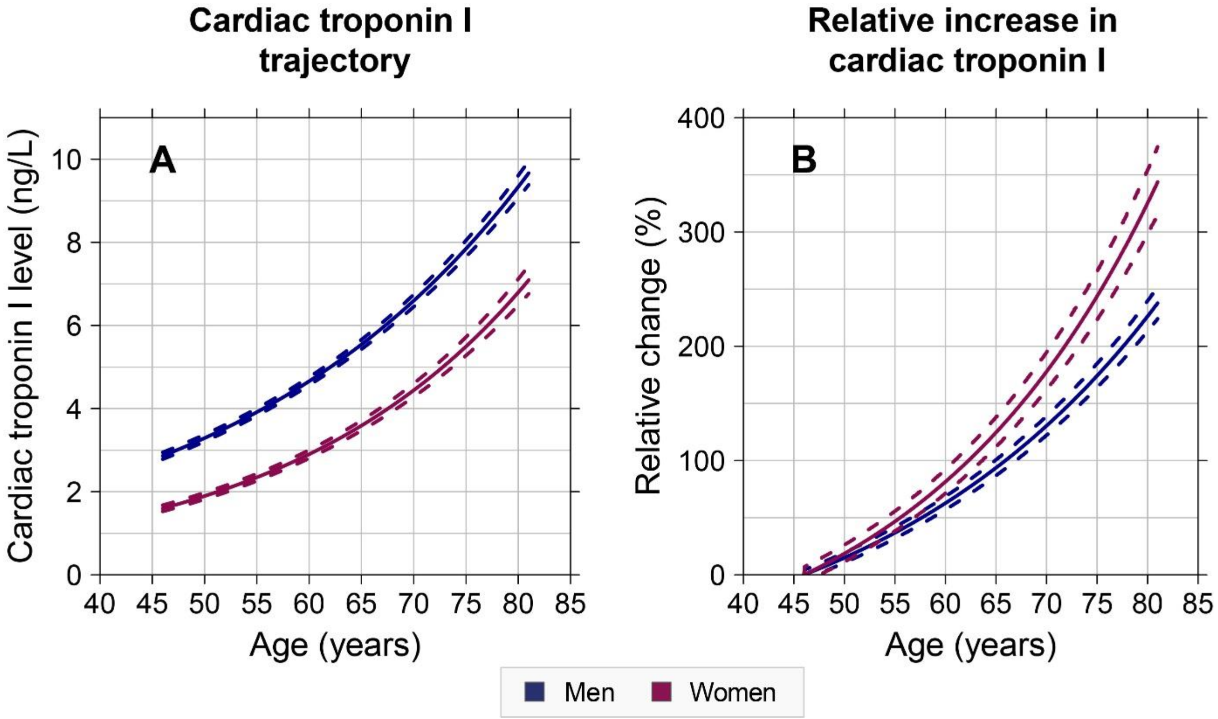

Supplemental Figure 2. Sex-specific cardiac troponin trajectories adjusted for known cardiovascular risk factors. Panel A depicts the sex-specific average trajectory of cardiac troponin over the middle to late adulthood life course. Panel B depicts the sex-specific average relative increase in cardiac troponin from 46 years onwards. The solid red line depicts the average trajectory of cardiac troponin in women and the solid blue line depicts the average trajectory of cardiac troponin in men. The dashed lines represent the 95% confidence intervals. Estimates are adjusted for ethnicity, diabetes mellitus, systolic blood pressure, total cholesterol, high-density lipoprotein, low-density lipoprotein, smoking status, and body mass index.

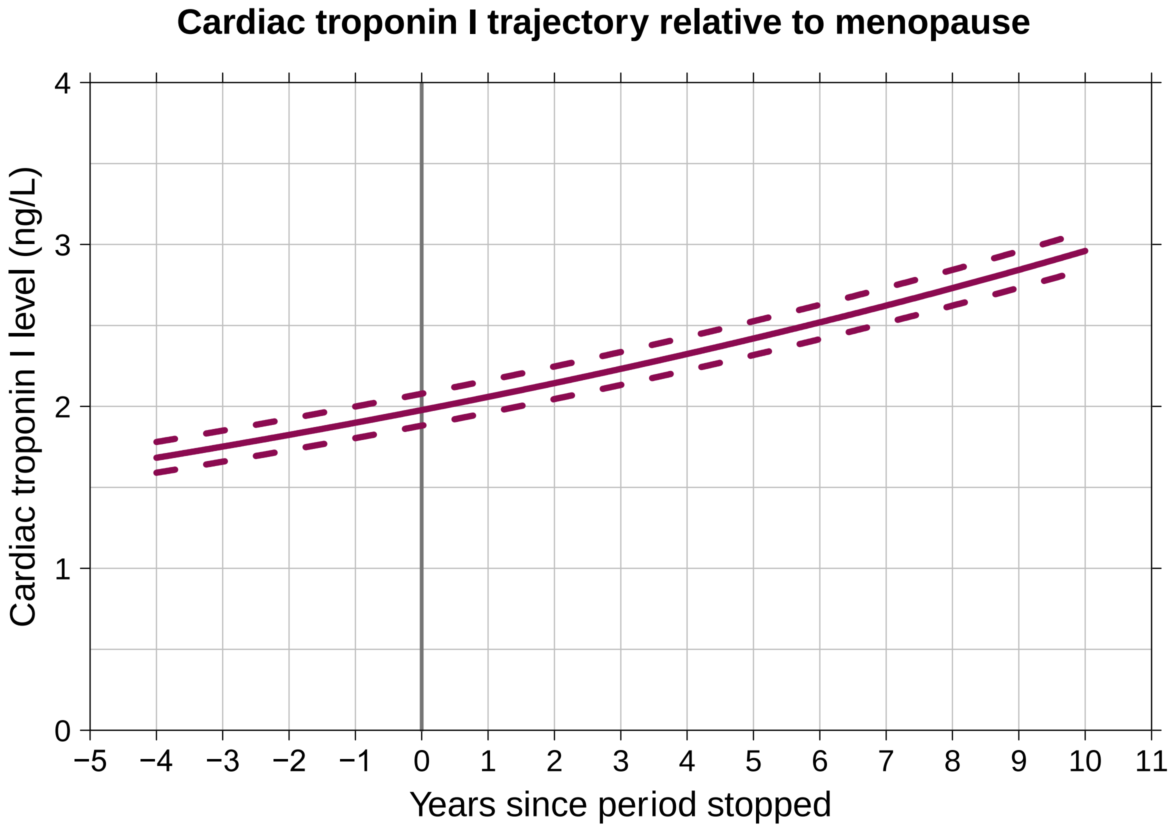

Supplemental Figure 3. Female-specific cardiac troponin trajectory relative to menopause. Information on the menopausal transition (i.e. age at which period stopped) was available for 1845 (86.1%) women. Women who reported that their period stopped as a result of hysterectomy (womb only), chemotherapy and/or radiation therapy were excluded from this analysis (19.2%). In total, 245 cardiac troponin measures were taken before menopause and 3127 after menopause.

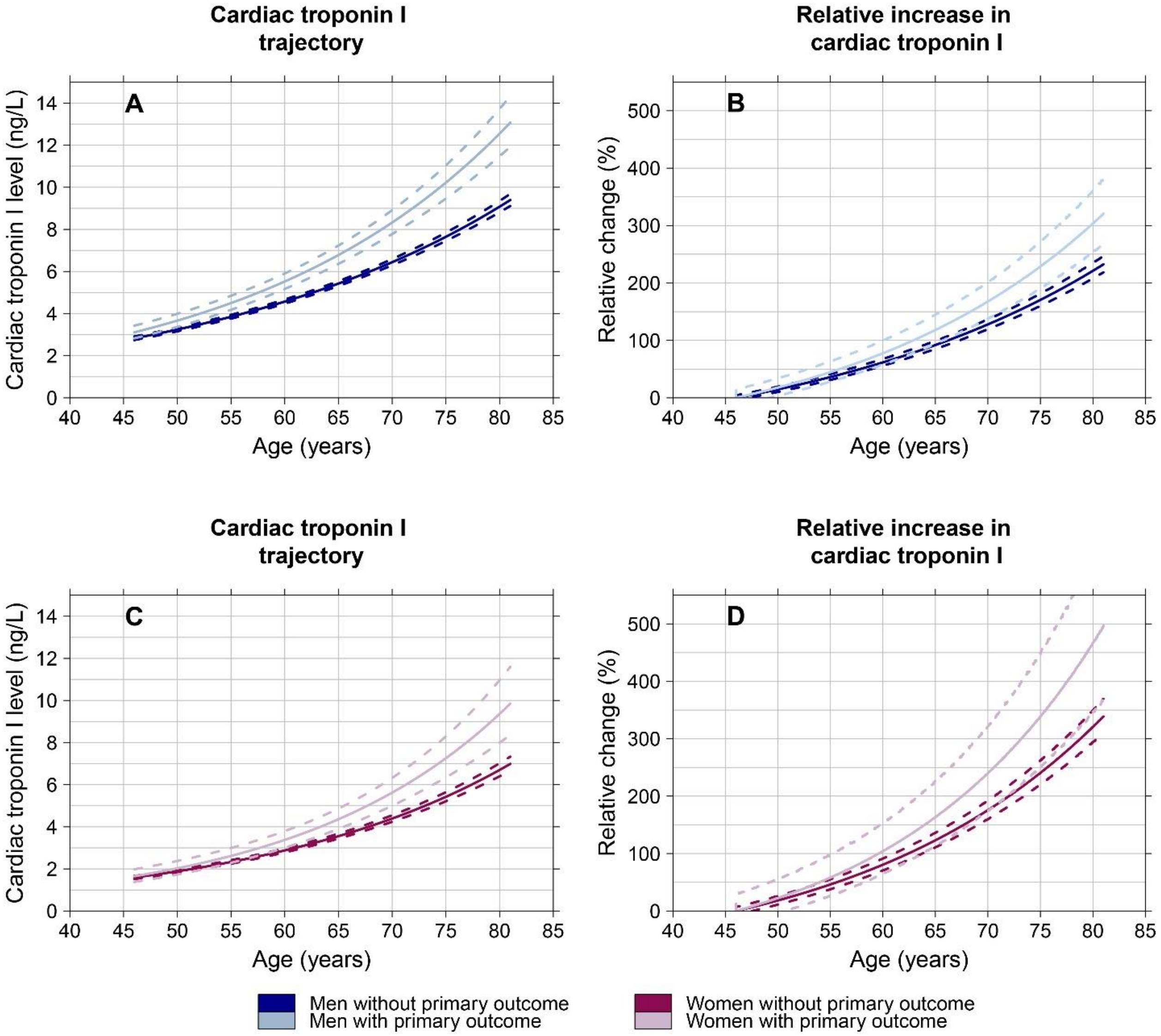

Supplemental Figure 4. Sex-specific cardiac troponin trajectories stratified by the primary outcome. Panels A and C depict the sex-specific average trajectory of cardiac troponin over the middle to late adulthood life course in those with and without the primary outcome (Panel A: men; Panel C: women). Panels B and D depict the sex-specific average relative increase in cardiac troponin from 46 years onward in those with and without the primary outcomes (Panel B: men; Panel D: women). The solid lines depict the average trajectory of cardiac troponin in a specific subgroup. The dashed lines represent the 95% confidence intervals.

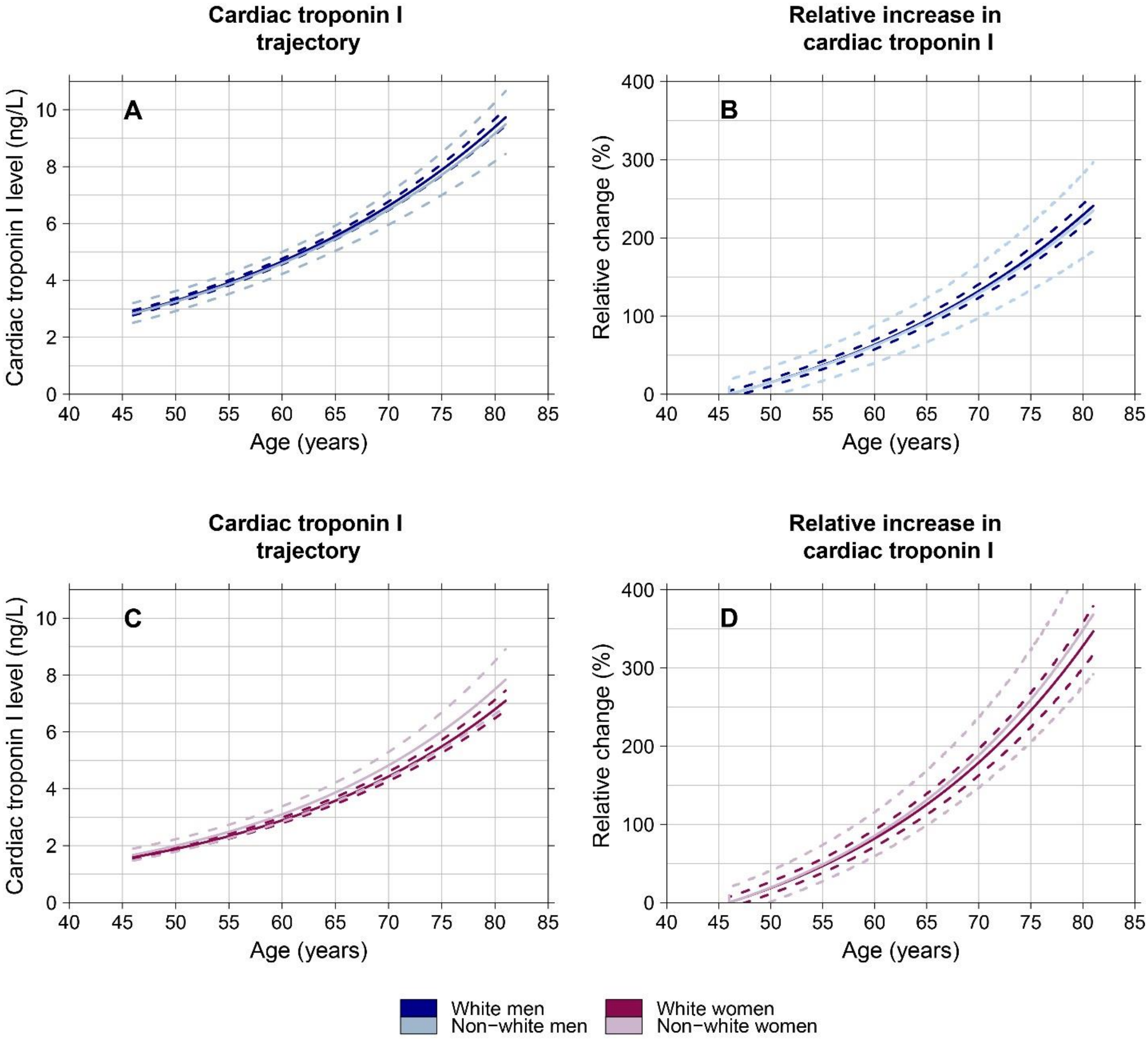

Supplemental Figure 5. Sex-specific cardiac troponin trajectories stratified by ethnicity. Panels A and C depict the sex-specific average trajectory of cardiac troponin over the middle to late adulthood life course stratified by ethnicity (Panel A: men; Panel C: women). Panels B and D depict the sex-specific average relative increase in cardiac troponin from 46 years onward stratified by ethnicity (Panel B: men; Panel D: women). The solid lines depict the average trajectory of cardiac troponin in a specific subgroup. The dashed lines represent the 95% confidence intervals.

Cumulative incidence of non-fatal myocardial infarction

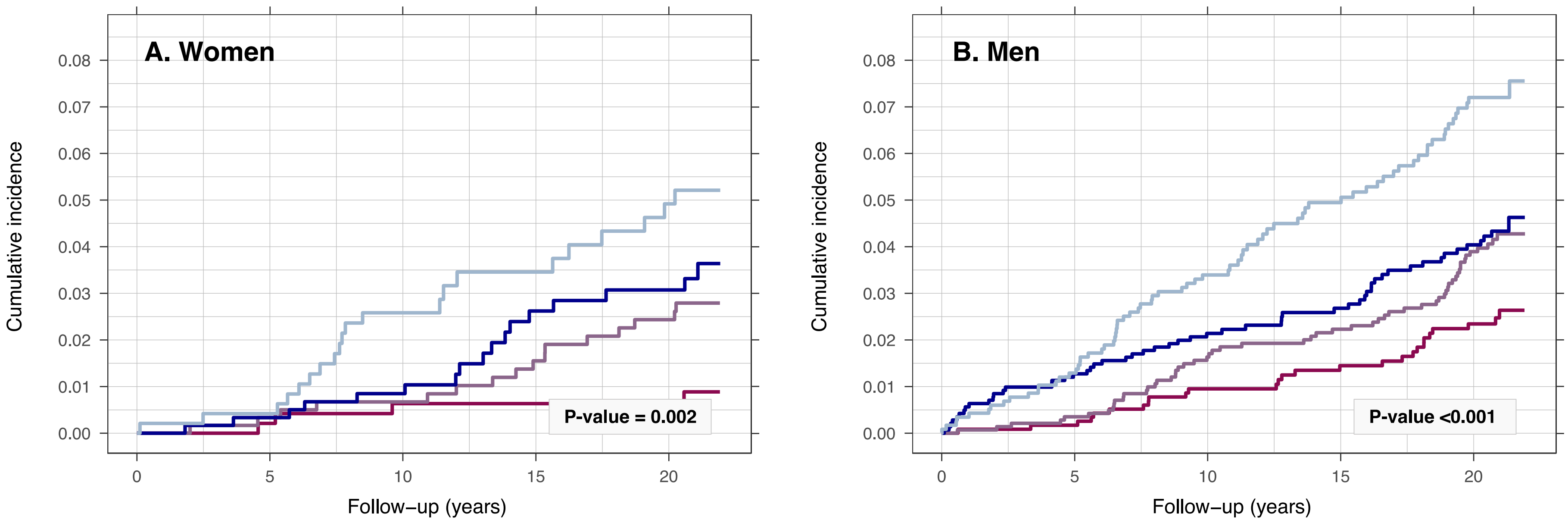

Group 1
Baseline cTnI level < median
and change in cTnI < median

Group 2
Baseline cTnI level < median
and change in cTnI ≥ median

Group 3
Baseline cTnI level ≥ median
and change in cTnI < median

Group 4
Baseline cTnI ≥ median
and change in cTnI ≥ median

Number at risk

Group 1	475	473	468	459	447	409	402	395	388
Group 2	596	593	585	571	558	523	512	498	482
Group 3	596	589	579	543	488	390	376	365	347
Group 4	475	471	461	427	401	303	290	267	249

Number at risk

Group 1	1162	1155	1143	1122	1086	953	929	910	870
Group 2	1413	1403	1393	1358	1317	1226	1205	1175	1127
Group 3	1413	1382	1351	1284	1197	970	942	912	877
Group 4	1163	1138	1111	1050	991	775	736	696	636

Supplemental Figure 6. Sex-specific association between non-fatal myocardial infarction and longitudinal cardiac troponin.

Cumulative incidence of non-fatal stroke

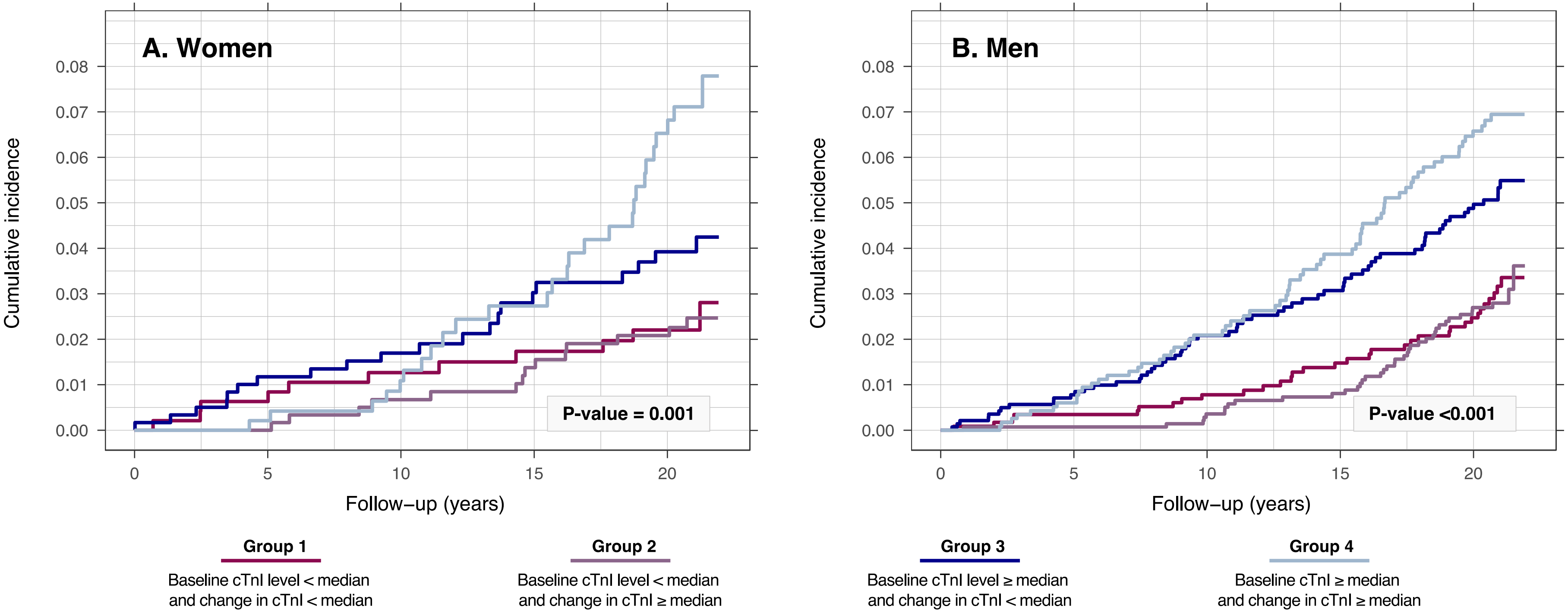

Number at risk (A. Women)

475	470	467	456	443	405	397	390	382
596	594	586	573	559	526	515	500	486
596	587	574	540	488	391	376	365	346
475	473	462	433	406	307	293	270	248

Number at risk (B. Men)

1162	1154	1143	1124	1090	954	929	907	869
1413	1404	1397	1368	1333	1238	1221	1185	1139
1413	1392	1362	1295	1203	976	947	919	881
1163	1142	1117	1065	1006	794	748	703	643

Supplemental Figure 7. Sex-specific association between non-fatal stroke and longitudinal cardiac troponin.

Cumulative incidence of cardiovascular death

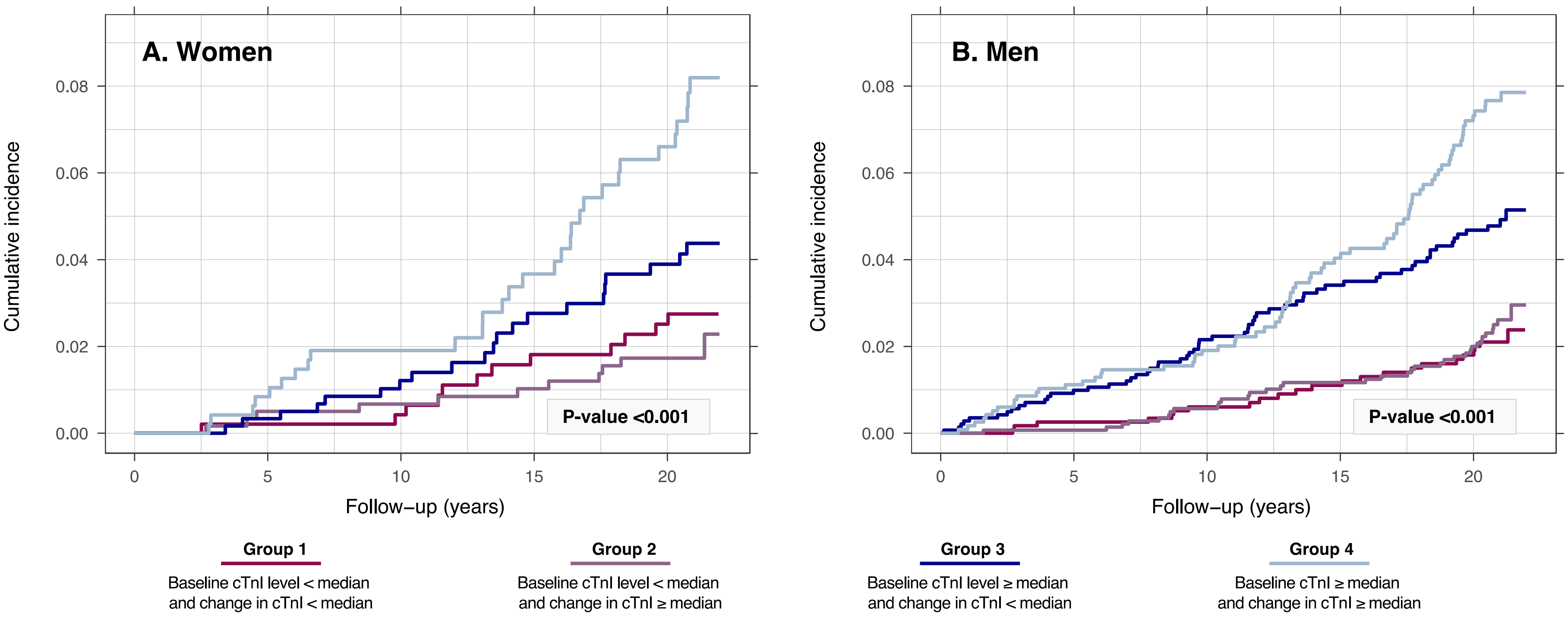

Number at risk (A. Women)

Group									
Group 1	475	474	469	460	448	409	402	396	388
Group 2	596	594	586	574	561	529	520	508	493
Group 3	596	590	581	547	493	395	385	373	356
Group 4	475	473	463	434	411	313	299	279	261

Number at risk (B. Men)

Group									
Group 1	1162	1156	1145	1128	1096	959	939	920	884
Group 2	1413	1405	1398	1368	1341	1247	1229	1204	1169
Group 3	1413	1396	1368	1305	1224	993	967	943	912
Group 4	1163	1144	1123	1077	1025	809	768	733	673

Supplemental Figure 8. Sex-specific association between cardiovascular death and longitudinal cardiac troponin.

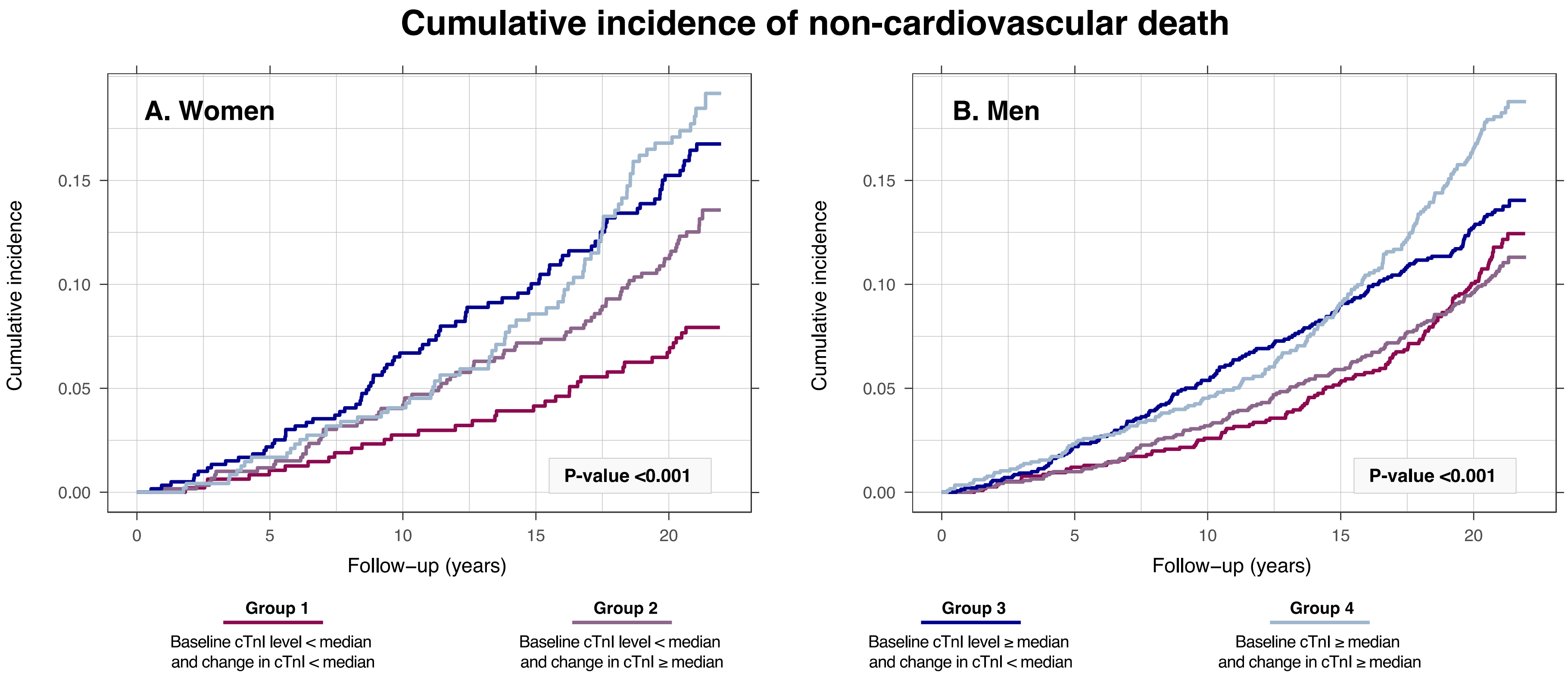

Number at risk

475	474	469	460	448	409	402	396	388
596	594	586	574	561	529	520	508	493
596	590	581	547	493	395	385	373	356
475	473	463	434	411	313	299	279	261

Number at risk

1162	1156	1145	1128	1096	959	939	920	884
1413	1405	1398	1368	1341	1247	1229	1204	1169
1413	1396	1368	1305	1224	993	967	943	912
1163	1144	1123	1077	1025	809	768	733	673

Supplemental Figure 9. Sex-specific association between non-cardiovascular death and longitudinal cardiac troponin.

The principle of joint modelling

To evaluate the association between a longitudinal marker (i.e., a measurement repeatedly monitored over time [cardiac troponin]), and the occurrence of an event over time (i.e. cardiovascular death), a joint model can be used. The joint model uses a separate regression model to describe the evolution of the marker over time and uses these estimated evolutions in a time-to-event relative risk model for the event of interest. A linear mixed-effects model is used to analyze the longitudinal marker over time, which results in an estimated level of the marker at each point in time, instead of assuming a constant level of the longitudinal marker between observed measurements. In the joint model, this estimated evolution is related to the event status (i.e., the estimated cardiac troponin I at the time of the event is used for the relative risk analysis). Apart from relating the level of the marker to the risk of the event, the joint modeling framework allows for extensions assessing additional associations. Perhaps it is not (only) the level of the marker that is related to the event but the fact that the marker is increasing rapidly at that moment. This would be of particular interest in situations where, for example, at a specific point in time two individuals show similar marker levels, but different rate of change of the marker. The rate of change of a longitudinal marker (i.e., the slope of the marker at that moment in time), can be added to the joint model to analyze its relationship with the event of interest. The slope, as evaluated by the joint model, indicates whether and by how much a marker is increasing or decreasing at any moment. In our study, we observed a significant relationship between the slope of cardiac troponin and the hazard of the primary outcome in women only (***Table 2***).

R code and educational material of linear mixed-effects models and multistate joint models

Simulating a demo dataset

The following R code simulates a dataset including a 1000 individuals (50% women, 50% men), that mimics data from the Whitehall II study. Namely, repeatedly measured cardiac troponin (ng/L) on the log2-scale, systolic blood pressure (mmHg), total cholesterol (mmol/L), low-denisty lipoprotien (mmol/L), high-density lipoprotein (mmol/L) and body mass index (kg/m2) are sampled at three time points per individual. Binary variables, diabetes (yes/no) and smoking status (yes/no), are sampled at one time point per individual in the simulated dataset, although these variables are repeatedly determined in the Whitehall II study. Ethnicity (white/non-white) is also sampled at one time point per individual.

```
set.seed(6)

# Helper functions to simulate longitudinal variables  -------------------------
simulate_variable <- function(beta0, beta1, beta2, beta3, beta4,
                              sd_randomintercept, sd_randomslope, residual_se){

 # Simulated longitudinal variable using pre-specified fixed and random effects
 # beta0, beta1, beta2, beta3, and beta3 are fixed effects
 # beta0 = intercept, beta1 = times, beta2 = sex, beta3 = age_baseline, beta4 = times:sex
 # sd_randomintercept, sd_randomslope, residual_se are used to simulate random effects

  # fixed effects coefficients
  fixed_effects <- c(beta0, beta1, beta2, beta3, beta4)

  # simulate random effects
  random_effects <- cbind(rnorm(n.total, sd = sd_randomintercept),
                          rnorm(n.total, sd = sd_randomslope))

  # determine linear predictor based on fixed and random effects
  eta.y <- as.vector(X %*% fixed_effects + rowSums(Z * random_effects[id, ]))

  # sample the longitudinal outcome values
  y <- rnorm(n.total * n.rep, eta.y, residual_se)

  return(y)
}
```

```
# Helper functions to simulate cardiac troponin  ----------------------------
simulate_troponin <- function(beta0, beta1, beta2, beta3, beta4, beta5, beta6, beta7,
                              beta8, beta9, beta10, beta11, beta12, beta13, beta14,
                              beta15, sd_randomintercept, sd_randomslope,
                              residual_se){

  set.seed(6)

  # Simulated longitudinal cardiac troponin using pre-specified fixed and random effects

  # design matrix fixed effects
  X <- model.matrix(~ 1 + times*sex + age_baseline*sex + diabetes*sex + tc + ldl + hdl +
                      sbp + smoking_status + bmi*sex + ethnicity, data = demo_dataset)

  # fixed effects coefficients
  fixed_effects <- c(beta0, beta1, beta2, beta3, beta4, beta5, beta6, beta7, beta8,
                     beta9, beta10, beta11, beta12, beta13, beta14, beta15)

  # simulate random effects
  random_effects <- cbind(rnorm(n.total, sd = sd_randomintercept),
                          rnorm(n.total, sd = sd_randomslope))

  # determine linear predictor based on fixed and random effects
```

```r
  eta.y <- as.vector(X %*% fixed_effects + rowSums(Z * random_effects[id, ]))

  # sample longitudinal cardiac troponin
  y <- rnorm(n.total * n.rep, eta.y, residual_se)

  return(y)
}
```

```r
# Demo dataset ------------------------------------------------------
# number of patients (women and men) --------------------------------
n.total <- 1000
proportion.women <- 0.50
n.women <- ceiling(proportion.women*n.total)
n.men <- n.total - n.women

# number of repeated measurements
n.rep <- 3

# initiate data frame to store demo_dataset --------------------------
id <- rep(1:n.total, each = n.rep)

# sample time-points
times <- replicate(n.total, c(0, sort(runif(n.rep - 1, min = 0.1, max = 15))),
                   simplify = FALSE) # minimum time = 0.1 years, maximum time = 15 years
times <- do.call(c, times)

# sample ages
age_baseline <- replicate(n.total, c(sort(runif( 1, min = 45, max = 80))),
                          simplify = FALSE) # minimum age = 45 years, maximum age = 80 years
age_baseline <- do.call(c, age_baseline)

# create dataframe
demo_dataset <- data.frame("id" = rep(1:n.total, each = n.rep), "times" = times,
                           "sex" = rep(rep(c("Male", "Female"), c(n.men, n.women)),
                                       each = n.rep),
                           "age_baseline" = rep(age_baseline, each = n.rep),
                           "age" = rep(age_baseline, each = n.rep) + times)

# design matrices for fixed and random effects  -----------------------------
X <- model.matrix(~ 1 + times*sex + age_baseline, data = demo_dataset)
Z <- model.matrix(~ 1 + times, data = demo_dataset)

# simulate (longitudinal) variables -----------------------------------------
# sbp (mmHg)
demo_dataset$sbp <- simulate_variable(beta0 = 94.46, beta1 = 0.34, beta2 = 2.11,
                                      beta3 = 0.48, beta4 = -0.04,
                                      sd_randomintercept = 12.89, sd_randomslope = 0.81,
                                      residual_se = 9.97)

# total cholesterol(mmol/L)
demo_dataset$tc <- 2^simulate_variable(beta0 = 2.91, beta1 = -0.01, beta2 = -0.054,
                                       beta3 = -0.006, beta4 = -0.008,
                                       sd_randomintercept = 0.21, sd_randomslope = 0.02,
                                       residual_se = 0.19)

# low-density lipoprotein (mmol/L)
demo_dataset$ldl <- 2^simulate_variable(beta0 = 2.55, beta1 = -0.02, beta2 = -0.001,
                                        beta3 = -0.01, beta4 = -0.01,
                                        sd_randomintercept = 0.30, sd_randomslope = 0.02,
                                        residual_se = 0.31)
```

```r
# high-denisty lipoprotein (mmol/L)
demo_dataset$hdl <- 2^simulate_variable(beta0 = 0.51, beta1 = 0.01, beta2 = -0.28,
                                       beta3 = 0.004, beta4 = -0.001,
                                       sd_randomintercept = 0.33, sd_randomslope = 0.02,
                                       residual_se = 0.15)

# bmi (kg/m2)
demo_dataset$bmi <- simulate_variable(beta0 = 24.11, beta1 = 0.05, beta2 = -0.46,
                                     beta3 = 0.04, beta4 = -0.01,
                                     sd_randomintercept = 3.94, sd_randomslope = 0.18,
                                     residual_se = 1.19)

# diabetes (yes/no)
demo_dataset$diabetes <- c(rep(sample(c("Yes", "No"), n.men, replace = T,
                                     prob = c(0.044, 0.956)), each = n.rep),# men
                           rep(sample(c("Yes", "No"), n.women, replace = T,
                                     prob = c(0.050, 0.950)), each = n.rep))# women

# smoking status (current smoker: yes/no)
demo_dataset$smoking_status <- c(rep(sample(c("Yes", "No"), n.men, replace = T,
                                           prob = c(0.085, 0.915)), each = n.rep),# men
                                 rep(sample(c("Yes", "No"), n.women, replace = T,
                                           prob = c(0.114, 0.886)), each = n.rep))# women

# ethnicity (white/other)
demo_dataset$ethnicity <- c(rep(sample(c("White", "Other"), n.men, replace = T,
                                      prob = c(0.936, 0.064)), each = n.rep),# men
                            rep(sample(c("White", "Other"), n.women, replace = T,
                                      prob = c(0.861, 0.139)), each = n.rep))# women

# log2_troponin (ng/L)
demo_dataset$log2_troponin <- simulate_troponin(beta0 = -1.80, beta1 = 0.06, beta2 = 1.07,
                                               beta3 = 0.04, beta4 = 0.12, beta5 = -0.1,
                                               beta6 = 0.07, beta7 = 0.20, beta8 = 0.004,
                                               beta9 = -0.03, beta10 = 0.004,
                                               beta11 = -0.05, beta12 = -0.012,
                                               beta13 = -0.011, beta14 = -0.18,
                                               beta15 = 0.011,
                                               sd_randomintercept = 1.0400,
                                               sd_randomslope = 0.0376,
                                               residual_se = 0.4197)
```

Example code: linear mixed-effects model used for Figure 1

We evaluated the temporal pattern of cardiac troponin in women and men over the middle to late adulthood life course using linear mixed-effects modeling. The distribution of cardiac troponin was skewed, and to achieve normal distribution we applied log2 transformation. Age was used as timescale and was entered as fixed effect and random effect in the model. Repeated measures of log2-transformed cardiac troponin were used as outcome.An interaction term for sex and age was used to estimate the average cardiac troponin trajectories in women and men separately.

```
# Sex-specific unadjusted linear mixed-effects model
lme.fit_unadjusted <- lme(log2_troponin ~ age*sex,
                          random = ~ age|id,
                          method = "REML",
                          data = demo_dataset,
                          control = lmeControl(opt='optim', msMaxIter = 150))
# Summary
summary(lme.fit_unadjusted)
```

```
## Linear mixed-effects model fit by REML
##   Data: demo_dataset
##        AIC      BIC    logLik
##   6903.457 6951.498 -3443.729
##
## Random effects:
##  Formula: ~age | id
##  Structure: General positive-definite, Log-Cholesky parametrization
##             StdDev     Corr
## (Intercept) 2.02950205 (Intr)
## age         0.02748987 -0.851
## Residual    0.44873567
##
## Fixed effects:  log2_troponin ~ age * sex
##                  Value  Std.Error   DF    t-value p-value
## (Intercept) -2.7634704 0.18221069 1998 -15.166346   0e+00
## age          0.0646524 0.00261576 1998  24.716516   0e+00
## sexMale      1.4508718 0.25621593  998   5.662692   0e+00
## age:sexMale -0.0132240 0.00369814 1998  -3.575868   4e-04
##  Correlation:
##             (Intr) age    sexMal
## age         -0.961
## sexMale     -0.711  0.683
## age:sexMale  0.679 -0.707 -0.960
##
## Standardized Within-Group Residuals:
##         Min          Q1         Med          Q3         Max
## -2.42000857 -0.55407480  0.02385866  0.54211280  2.57605567
##
## Number of Observations: 3000
## Number of Groups: 1000
```

Example code: linear mixed-effects model used for Figure 2A and Figure 2B

Univariable and multivariable sex-specific linear mixed-effects models were used to evaluate the association of ethnic origin and repeatedly determined diabetes, systolic blood pressure, total cholesterol, high-density lipoprotein, low-density lipoprotein, smoking status, body mass index with repeatedly measured cardiac troponin. Repeated measures of the cardiovascular risk factors, assessed at time of cardiac troponin sampling, were entered in the models as fixed effects while repeated log2-transformed cardiac troponin measures were used as outcome. An interaction term for sex and the risk factor of interest was used to estimate associations with cardiac troponin in women and men separately.
Note: in the demo dataset, diabetes and smoking status were sample at baseline.

```r
# Sex-specific univariable linear mixed-effects model (variable of interest = ethnicity)
lme.fit_univariable <- lme(log2_troponin ~ ethnicity*sex,
                       random = ~ 1|id,
                       method = "REML",
                       data = demo_dataset,
                       control = lmeControl(opt='optim', msMaxIter = 150))
# Summary
summary(lme.fit_univariable)
```

```
## Linear mixed-effects model fit by REML
##   Data: demo_dataset
##        AIC      BIC    logLik
##   7937.398 7973.428 -3962.699
##
## Random effects:
##  Formula: ~1 | id
##         (Intercept)  Residual
## StdDev:    1.192944 0.5879198
##
## Fixed effects:  log2_troponin ~ ethnicity * sex
##                             Value Std.Error   DF   t-value p-value
## (Intercept)             1.6446517 0.1471959 2000 11.173213  0.0000
## ethnicityWhite          0.0133327 0.1589104  996  0.083901  0.9332
## sexMale                 0.5422232 0.2414407  996  2.245782  0.0249
## ethnicityWhite:sexMale -0.0495351 0.2554178  996 -0.193938  0.8463
##  Correlation:
##                        (Intr) ethncW sexMal
## ethnicityWhite         -0.926
## sexMale                -0.610  0.565
## ethnicityWhite:sexMale  0.576 -0.622 -0.945
##
## Standardized Within-Group Residuals:
##         Min          Q1         Med          Q3         Max
## -3.10126887 -0.55065170 -0.02919807  0.53951578  3.07399602
##
## Number of Observations: 3000
## Number of Groups: 1000
```

```
# Sex-specific multivariable linear mixed-effects model
# (variables of interest = age, ethnicity, diabetes, systolic blood pressure,
# total cholesterol, low-density lipoprotein, high-density lipoprotein, smoking status
# and body mass index)
lme.fit_multivariable <- lme(log2_troponin ~ (age + ethnicity + diabetes + sbp + tc +
                                               hdl + ldl + smoking_status + bmi)*sex,
                        random = ~ 1|id,
                        method = "REML",
                        data = demo_dataset,
                        control = lmeControl(opt='optim', msMaxIter = 150))

# Summary
summary(lme.fit_multivariable)
```

```
## Linear mixed-effects model fit by REML
##   Data: demo_dataset
##        AIC      BIC    logLik
##   6925.476 7057.469 -3440.738
##
## Random effects:
##  Formula: ~1 | id
##         (Intercept)  Residual
## StdDev:    1.081042 0.4679231
##
## Fixed effects:  log2_troponin ~ (age + ethnicity + diabetes + sbp + tc + hdl +      ldl + smoking_status +
##                                Value Std.Error   DF   t-value p-value
## (Intercept)               -2.1672467 0.3460379 1988 -6.263032  0.0000
## age                        0.0634478 0.0025679 1988 24.707699  0.0000
## ethnicityWhite            -0.0738492 0.1429346  992 -0.516664  0.6055
## diabetesYes                0.2165931 0.1986738  992  1.090195  0.2759
## sbp                       -0.0003577 0.0012683 1988 -0.282062  0.7779
## tc                        -0.0933438 0.0160818 1988 -5.804318  0.0000
## hdl                        0.0309487 0.0540001 1988  0.573123  0.5666
## ldl                        0.0376188 0.0161541 1988  2.328747  0.0200
## smoking_statusYes          0.0322894 0.1505710  992  0.214446  0.8302
## bmi                       -0.0032071 0.0073677 1988 -0.435294  0.6634
## sexMale                    0.7076014 0.5147338  992  1.374694  0.1695
## age:sexMale               -0.0166542 0.0037872 1988 -4.397458  0.0000
## ethnicityWhite:sexMale     0.0005581 0.2303775  992  0.002423  0.9981
## diabetesYes:sexMale       -0.3726390 0.3105624  992 -1.199884  0.2305
## sbp:sexMale                0.0044960 0.0017800 1988  2.525929  0.0116
## tc:sexMale                -0.0156038 0.0233518 1988 -0.668207  0.5041
## hdl:sexMale                0.0845443 0.0855453 1988  0.988298  0.3231
## ldl:sexMale                0.0377696 0.0230639 1988  1.637608  0.1017
## smoking_statusYes:sexMale  0.1229780 0.2402989  992  0.511771  0.6089
## bmi:sexMale                0.0082322 0.0103895 1988  0.792362  0.4282
##  Correlation:
##                           (Intr) age    ethncW dbtsYs sbp    tc     hdl
## age                       -0.375
## ethnicityWhite            -0.364 -0.021
## diabetesYes               -0.011 -0.026 -0.020
## sbp                       -0.375 -0.186 -0.004  0.030
## tc                        -0.353  0.219  0.006 -0.014 -0.036
## hdl                       -0.154 -0.219  0.020 -0.031  0.013 -0.027
## ldl                       -0.296  0.213  0.012  0.012  0.039  0.033  0.006
## smoking_statusYes         -0.048  0.034 -0.019  0.055  0.000  0.007 -0.018
## bmi                       -0.506 -0.127  0.025 -0.024  0.005 -0.016 -0.032
## sexMale                   -0.672  0.252  0.245  0.008  0.252  0.237  0.103
## age:sexMale                0.254 -0.678  0.015  0.018  0.126 -0.149  0.149
## ethnicityWhite:sexMale     0.226  0.013 -0.620  0.013  0.002 -0.004 -0.012
## diabetesYes:sexMale        0.007  0.017  0.013 -0.640 -0.019  0.009  0.020
```

```
## sbp:sexMale                0.267  0.133  0.003 -0.021 -0.713  0.026 -0.009
## tc:sexMale                 0.243 -0.151 -0.004  0.010  0.025 -0.689  0.019
## hdl:sexMale                0.097  0.138 -0.012  0.020 -0.008  0.017 -0.631
## ldl:sexMale                0.207 -0.149 -0.008 -0.009 -0.027 -0.023 -0.004
## smoking_statusYes:sexMale  0.030 -0.022  0.012 -0.034  0.000 -0.004  0.011
## bmi:sexMale                0.359  0.090 -0.018  0.017 -0.004  0.011  0.023
##                           ldl    smkn_Y bmi    sexMal ag:sxM ethW:M dbtY:M
## age
## ethnicityWhite
## diabetesYes
## sbp
## tc
## hdl
## ldl
## smoking_statusYes         -0.006
## bmi                       -0.030 -0.027
## sexMale                    0.199  0.032  0.340
## age:sexMale               -0.144 -0.023  0.086 -0.403
## ethnicityWhite:sexMale    -0.007  0.012 -0.016 -0.406 -0.018
## diabetesYes:sexMale       -0.008 -0.035  0.015 -0.008 -0.029 -0.003
## sbp:sexMale               -0.028  0.000 -0.004 -0.355 -0.173 -0.006  0.004
## tc:sexMale                -0.022 -0.005  0.011 -0.383  0.284 -0.003 -0.011
## hdl:sexMale               -0.004  0.012  0.020 -0.181 -0.212  0.004  0.003
## ldl:sexMale               -0.700  0.004  0.021 -0.303  0.262  0.008  0.004
## smoking_statusYes:sexMale  0.004 -0.627  0.017 -0.084  0.019  0.054  0.060
## bmi:sexMale                0.021  0.019 -0.709 -0.485 -0.130  0.027 -0.023
##                           sbp:sM tc:sxM hdl:sM ldl:sM sm_Y:M
## age
## ethnicityWhite
## diabetesYes
## sbp
## tc
## hdl
## ldl
## smoking_statusYes
## bmi
## sexMale
## age:sexMale
## ethnicityWhite:sexMale
## diabetesYes:sexMale
## sbp:sexMale
## tc:sexMale                -0.022
## hdl:sexMale                0.018  0.012
## ldl:sexMale                0.017  0.009  0.021
## smoking_statusYes:sexMale  0.004  0.011 -0.004 -0.004
## bmi:sexMale                0.001 -0.009  0.010 -0.017  0.005
##
## Standardized Within-Group Residuals:
##         Min          Q1         Med          Q3         Max
## -2.82944927 -0.54859057  0.01761317  0.52245323  2.95552539
##
## Number of Observations: 3000
## Number of Groups: 1000
```

Example code: linear mixed-effects model used for Figure 3 and Figure 4

We estimated the sex-specific baseline cardiac troponin level and sex-specific slope parameter for each individual using linear mixed-effects modeling. Age was used as timescale and was entered as fixed effect and random effect in the model. Repeated measures of log2-transformed cardiac troponin were used as outcome. An interaction term for sex and age was used to estimate the cardiac troponin trajectories in women and men separately. Individual's cardiac troponin level at baseline was estimated for age at baseline using predict function and individual's slope parameters were extracted from the linear mixed-effects model. To extract sex-specific slope parameters, models were fitted with both female sex and male sex as a reference level.

```
# Linear mixed-effects model with female sex as reference level
lme.fit <- lme(log2_troponin ~ age*sex,
               random = ~ age|id,
               method = "REML",
               data = demo_dataset,
               control = lmeControl(opt='optim', msMaxIter = 150))

# Refit model with male sex as reference level
demo_dataset$sex_reverse <-  factor(demo_dataset$sex, levels = c("Male", "Female"))

lme.fit_reverse <- lme(log2_troponin ~ age*sex_reverse,
                     random = ~ age|id,
                     method = "REML",
                     data = demo_dataset,
                     control = lmeControl(opt='optim', msMaxIter = 150))

# Extracting individual sex-specific slopes and baseline levels from linear mixed-effects
# models
df_trajectory <- data.frame("id" = row.names(random.effects(lme.fit)),
                 "sex" = demo_dataset$sex,
                 "individual_slopes"=ifelse(row.names(random.effects(lme.fit)) %in% unique(
                              demo_dataset[demo_dataset$sex == "Female", "id"]),
                                                         coef(lme.fit)[["age"]],
                                                         coef(lme.fit_reverse)[["age"]]),
                 "individual_baselinelevel" = predict(lme.fit,
                                              newdata = demo_dataset[demo_dataset$times == 0,
                                              c("id", "sex", "age")],
                                              level = 0:1)[["predict.id"]])
```

Subsequently, we determined the sex-specific cut-off values for baseline level and slope (i.e., sex-specific medians) and, based on the results, classified individuals into the following four groups:

- Group 1 = baseline level $<$ sex-specific median and change $<$ sex-specific median
- Group 2 = baseline level $<$ sex-specific median and change $\geq$ sex-specific median
- Group 3 = baseline level $\geq$ sex-specific median and change $<$ sex-specific median
- Group 4 = baseline level $\geq$ sex-specific median and change $\geq$ sex-specific median

```
df_trajectory %>% group_by(sex) %>%
  summarize("median_baselinelevel" = median(2^individual_baselinelevel))
```

```
## # A tibble: 2 x 2
##   sex    median_baselinelevel
##   <chr>                 <dbl>
## 1 Female                 2.91
## 2 Male                   3.32
```

```
df_trajectory %>% group_by(sex) %>%
  summarize("median_slope" = median(2^individual_slopes)) # ~ 4% per year
```

```
## # A tibble: 2 x 2
##   sex    median_slope
##   <chr>         <dbl>
## 1 Female         1.04
## 2 Male           1.04
```

Multistate joint models

We applied sex-specific multistate joint modeling to evaluate the association between individual cardiac troponin trajectories and cardiovascular events in women and men separately using the JMbayes2 package. The application of joint models with and without handling competing risk are nicely illustrated on the website of Professor Dimitris Rizopoulos (Erasmus University, Rotterdam, The Netherlands), author of the JMbayes2 package (https://drizopoulos.github.io/JMbayes2/index.html).

The original R code for this study is available upon request from the corresponding author.

Corresponding author:
Dorien M Kimenai, PhD
BHF/University Centre for Cardiovascular Science
The University of Edinburgh
Edingburgh EH16 4SA
United Kingdom
Email: dorien.kimenai@ed.ac.uk
